# Validating ICD code case definitions for condition case ascertainment in multimorbidity measurement: a retrospective chart review

**DOI:** 10.1093/gerona/glag157

**Published:** 2026-06-10

**Authors:** Ashley J Kang, Chi-Hong Tseng, Melissa Y Wei

**Affiliations:** Division of General Internal Medicine and Health Services Research, Department of Medicine, University of California at Los Angeles, Los Angeles, California, United States; Division of General Internal Medicine and Health Services Research, Department of Medicine, University of California at Los Angeles, Los Angeles, California, United States; Division of General Internal Medicine and Health Services Research, Department of Medicine, University of California at Los Angeles, Los Angeles, California, United States; Center for the Study of Healthcare Innovation, Implementation and Policy, VA Greater Los Angeles Healthcare System, Los Angeles, California, United States; (Medical Sciences Section)

**Keywords:** Chronic conditions, Multimorbidity, Comorbidity, Chart review, Electronic health record data

## Abstract

**Background:**

Accurate case ascertainment of chronic conditions is critical for research, clinical decision-making, and population health management, especially for older adults with high multimorbidity. However, validation of International Classification of Diseases (ICD)-based case definitions in the electronic health record (EHR) data remains limited. We sought to determine whether a single ICD code is sufficient for accurate identification of chronic conditions in the EHR, or whether multiple codes improve validity.

**Methods:**

Population-based retrospective chart review, 2013-2019, at large academic tertiary and quaternary care health system. Our sample included adults aged ≥18 years with ≥2 encounters within a 2-year period. We conducted gold-standard chart review to determine validity of using ≥1 or ≥2 ICD codes to identify 23 chronic conditions included in the validated multimorbidity-weighted index, specifically those lacking robust case definitions in EHR data. Validation statistics included positive predictive value (PPV), negative predictive value, Cohen’s kappa, sensitivity, specificity, and percent sample size loss associated with stricter ≥2 ICD code case definitions.

**Results:**

The final analytic sample included 780 873 adults (mean [SD] age 47.7 [18.0] years, 56.9% female). For 22 of 23 conditions, use of ≥1 ICD code yielded a PPV ≥0.70. Requiring ≥2 ICD codes yielded minimal improvement in PPV but with substantial sample size loss that ranged from 16.8% to 64.5%.

**Conclusions:**

At least one ICD code was sufficient to accurately identify most chronic conditions with high accuracy in the EHR over a multi-year timeframe. However, condition-specific validation remains essential, as performance varies by condition.

## Introduction

Accurate ascertainment of chronic conditions is fundamental for clinical epidemiology and health services research, including the evaluation of individual conditions or several coexisting chronic conditions, in the case of multimorbidity and comorbidity highly prevalent in older and aging adults. It also plays a critical role in clinical practice, such as complex case management, where risk stratification tools routinely incorporate measures of chronic condition burden.^1^^,^^2^

Currently, the Centers for Medicare and Medicaid Services Chronic Conditions Data Warehouse (CCW)^3^ case definitions are among the most widely used approaches for disease ascertainment. These definitions rely on the number of International Classification of Diseases (ICD) codes, type of claim (eg, inpatient, outpatient), within a defined reference period. For 24 of 30 CCW conditions, the condition case definition is the widely used one inpatient or two outpatient ICD codes within 2 years. However, many of the reference studies^4^ backing these case definitions lack explicit validation against a gold standard.

Furthermore, the CCW definitions were not developed for electronic health record (EHR) data systems that are now widespread among US office-based physicians and hospitals.^5^ Electronic health record data fundamentally differ from claims data in both purpose and scope.^6^ Electronic health record data also often captures a broader, more diverse population with different sociodemographic characteristics and healthcare utilization patterns. Many CCW-based studies were conducted in older, insured Medicare beneficiaries,^4^ limiting their generalizability. Furthermore, while older adults have the highest burden of multimorbidity,^7^ the accumulation of chronic conditions in younger and middle-aged adults is equally important to capture and assess, as multimorbidity progression throughout the lifespan is upstream to outcomes such as frailty and functional decline in later life.^8^

Therefore, as EHR data have become increasingly available and central to research, prior studies have extended this work of developing case definitions to the EHR. These studies commonly examine the range of accuracy statistics (eg, positive predictive value [PPV]) of ICD codes for various chronic conditions. Prior studies suggest that requiring more ICD codes may improve PPV but often at the cost of reduced sensitivity and sample size. Importantly, the magnitude of this tradeoff varies by condition.^9–12^ To date, work has focused on validating EHR-based ICD case definitions for a limited number of individual chronic conditions. To address this gap, we examined the performance of both at least 1 (≥1) and at least 2 (≥2) ICD code definitions across a broad inventory of chronic conditions in a comprehensive, validated measure of multimorbidity that includes all chronic CCW conditions plus others. Specifically, we assessed all the chronic conditions in the multimorbidity-weighted index (MWI)^13–17^ without existing validated EHR-based case definitions and quantified the sample size impact when using the stricter ≥2 ICD codes case definition compared with ≥1 ICD code.

We conducted this study in the University of California, Los Angeles (UCLA) Health System, which serves a large, sociodemographically diverse patient population across Los Angeles County. Using several years of EHR data, we aimed to provide a rigorous, comprehensive assessment of ICD-based chronic condition definitions and to evaluate the generalizability of commonly used claims-based CCW definitions in EHR data. More accurate case ascertainment of chronic conditions will inform robust multimorbidity and comorbidity research, strengthen individual condition analyses, and support more robust risk stratification and clinical decision support.

## Methods

### Overview

Using UCLA EHR data, we conducted chart review to validate ICD-based case definitions for a set of chronic conditions not previously validated in EHR data.

### Study population: UCLA EHR

We created a closed cohort of adults aged ≥18 years with continuity care at UCLA Health between 2013 and 2019 as the initial sample for chart-review eligibility to validate ICD-based case definitions. UCLA Health is a tertiary and quaternary academic health system serving diverse patient populations across clinics at UCLA and surrounding regions. Epic (Epic Systems Corporation),^18^ the EHR platform used by UCLA Health, contains patient demographics, clinical, appointment, and billing data from UCLA Health System encounters and available outside records.

For inclusion, participants must have been 18 years old in 2013 and have ≥2 encounters documented in the EHR (ie, outpatient visits, hospitalizations, home-based primary care, telemedicine) within a 2-year period between 2013 and 2019. UCLA Health implemented its current EHR system in 2013,^19^ and we used data through 2019 to capture the 7-year period preceding the COVID-19 pandemic. We include available baseline demographic characteristics. This study was approved by the UCLA Institutional Review Board (IRB no. 22-000366 and 23-001574).

### Chronic condition assessment

We assessed conditions in the MWI^13–17^ that lacked validated case definitions in the EHR based on literature review. MWI is a person-centered, comprehensive measure of multimorbidity that weights conditions by their impact on physical functioning. MWI includes chronic conditions spanning all organ systems, inpatient and outpatient care settings, age groups across the lifespan, and sex-specific diagnoses. Guided by literature searches, we identified 23 unique, impactful MWI conditions without prior EHR-based definitions and assessed the performance of ICD codes for these conditions through chart review. The remaining MWI conditions with existing EHR-based definitions, including hypertension, hypothyroidism, osteoarthritis, anxiety, and depression, among others (full list of MWI conditions in Appendix Table S1) were not assessed in the present study.

The 23 chronic conditions assessed include: anemia; aortic aneurysm; Barrett’s esophagus; blood cancers; calculus of kidney, ureter; cataract; cervical cancer; colon polyp; disk disorders; dysmenorrhea; erectile dysfunction; hepatitis, hepatocellular disease; hyperthyroidism; interstitial cystitis; liver cancer; osteoporosis; other cancers; other neurologic disorders; pancreatitis; peptic ulcer; premenstrual syndrome (PMS), premenstrual dysphoric disorder; restless legs syndrome; substance use disorders. “Other cancers” and “other neurologic disorders” include oncologic and neurologic conditions not already categorized separately in MWI (eg, Huntington’s disease; see Appendix File S1).

### ICD-based case definitions

We evaluated the performance of ICD-based case definitions for the 23 chronic conditions and condition groups without prior validation in the EHR. We assessed the performance of ≥1 ICD code for the condition as well as the performance of ≥2 ICD codes for the condition.

### Chart review sample

For each of the 23 chronic conditions, we identified all UCLA Health patients with ≥1 and ≥2 ICD codes for the condition anytime during the study timeframe, across all encounter settings. Codes were adopted from MWI-specific inventories of relevant ICD-9-CM and ICD-10-CM codes for a given condition, to cover diagnoses in the pre- and post-2015 ICD transition. Our MWI condition ICD-10-CM codes were mapped from previously validated ICD-9-CM code lists,^16^ and mapping validation was done in UCLA data.^17^ For each condition, chart review of “cases” was conducted on a random sample of those with ≥1 ICD code and a random sample of those with ≥2 ICD codes, totaling 40 patient charts for each condition (20 patients with ≥1 ICD code, 20 patients with ≥2 ICD codes). In total, we reviewed 920 total “case” charts (23 conditions × 40 charts).

To assess false negatives, we created a “controls” cohort of 50 randomly selected charts (from the total pool of chart review “cases”) and assessed for the presence of all 23 chronic conditions for which the patient had no ICD codes during the study period. To evaluate sex-specific diagnoses, we additionally randomly selected and reviewed 25 female and 25 male charts to supplement this assessment for sex-specific conditions, since patient charts were not assessed for sex-inapplicable conditions. In total, the “controls” review included 1002 unique chart-condition combinations, averaging 44 assessments per condition.

### Chart review condition case ascertainment protocol

We conducted gold-standard chart review in the UCLA EHR to assess performance of ICD-based case definitions. For each of the 23 conditions, we developed chart review protocols to determine whether charts were “case positive” (evidence supported the diagnosis) or “case negative” (insufficient evidence). Protocol development was led by a primary care physician (M.Y.W.) and final protocols were refined through pilot testing. Full protocol details are in Appendix File S1. For all condition protocols, review began with objective data domains, including pathology and imaging reports, medications, and laboratory results. We also reviewed primary care, relevant specialist, hospitalization, and emergency department notes. A chart was deemed “case positive” if relevant objective data or clinical documentation confirmed the diagnosis. Otherwise, the chart was assigned “case negative” for insufficient evidence to support the diagnosis.

### Chart reviewers

Chart review was conducted by trained abstractors (including project manager A.J.K., a research assistant, and nurse abstractor) using protocols developed for each condition. For charts reviewed by two abstractors, disagreement was resolved by a third trained arbitrator and physician on the team (M.Y.W.).

### Validation statistics to evaluate ICD-based case definitions

We calculated PPV for the ≥1 and ≥2 ICD code case definitions for each condition. Negative predictive value (NPV) was calculated from chart-condition combinations in patients without any ICD codes indicating the condition. We also calculated sensitivity and specificity for the ≥1 code case definitions. To assess inter-rater reliability (agreement), we calculated Cohen’s kappa (κ). Fifty percent of all charts were evaluated by both abstractors. Final PPV and NPV were calculated after discrepancies were resolved by a third reviewer. We computed 95% confidence intervals (CI) for all statistics. Finally, to evaluate sample size implications, we quantified the percentage of patients lost when applying the stricter ≥2 ICD code definition compared to the broader ≥1 code definition.

## Results

### Participant characteristics

The final sample eligible for chart review included 780 873 adults with ≥2 encounters at UCLA Health, with a mean age of 47.7 (SD 18.0) in 2013 (Table 1). Participants were 56.9% female, 43.8% non-Hispanic White, 4.6% non-Hispanic Black, 9.0% non-Hispanic Asian, and 12.1% Hispanic/Latino, with mean social vulnerability index (SVI) of 0.4 (SD 0.3). The CDC’s SVI quantifies a community’s social vulnerability from 0 to 1 (least-most vulnerable).^20^ Participants had a mean MWI score of 5.1 (SD 7.7) using encounter diagnoses from 2013-2019.

**Table 1:** Participant characteristics of UCLA Health adults eligible for chart review at baseline,^a^  *N* = 780 873.

	**Mean** **±** **SD or *N* (%)**
**Age in 2013, years**	47.68 ± 17.97
**Sex, female**	444 592 (56.94)
**Race/ethnicity**	
Non-Hispanic White	342 290 (43.83)
Non-Hispanic Black	36 202 (4.64)
Non-Hispanic Asian	70 306 (9.00)
Hispanic/Latino of any race	94 402 (12.09)
Other/unknown	237 673 (30.44)
**Social vulnerability index^b^**	0.40 ± 0.27
**Multimorbidity-weighted index, 2013-2019**	5.14 ± 7.74

Abbreviations: MWI, multimorbidity-weighted index; SD, standard deviation; SVI, Social Vulnerability Index.

a All patient characteristics measured at baseline with exception of SVI (most recent value available) and MWI (cumulative score based on all diagnoses from baseline through 2019).

b SVI ranges from 0 (*least vulnerable*) to 1 (*most vulnerable*).^20^

### PPV for ≥1 ICD code

**Figure 1:**
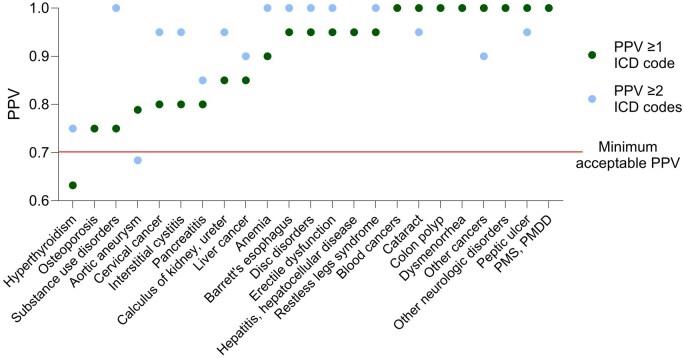
PPV of ≥1 and ≥2 ICD codes for 23 MWI chronic conditions and condition groups assessed in chart review, UCLA Health EHR, 2013-2019. Only one condition, hyperthyroidism, did not meet a priori acceptable threshold of PPV ≥ 0.70 when using ≥1 code as the case definition. ICD, International Classification of Diseases; PMDD, premenstrual dysphoric disorder; PMS, premenstrual syndrome; PPV, positive predictive value.

When requiring ≥1 ICD code, 22 of 23 chronic conditions demonstrated a PPV that exceeded the a priori acceptable threshold of 0.70.^21–26^ The exception was hyperthyroidism, which had a PPV of 0.63. The mean PPV across all conditions was 0.90 (range 0.63-1.0; Table 2, Figure 1).

**Table 2:** Validation statistics for 23 MWI chronic conditions assessed by gold standard chart review in the UCLA Health EHR, 2013-2019.

Condition	**PPV** **≥** **1**	**PPV** **≥** **2**	Absolute change in PPV	NPV	Cohen’s kappa	**Number of unique patients with** **≥** **1 ICD code**	**Number of unique patients with** **≥** **2 ICD code**	**% cases lost if using ≥** **2 vs ≥** **1 code case definition**
**Anemia**	0.9	1	0.10	1	1	89 257	60 678	32.02
**Aortic aneurysm (aortic aneurysm and dissection)**	0.79	0.68	0.11	1	1	6504	4684	27.98
**Barrett’s esophagus**	0.95	1	0.05	1	0.91	3065	2010	34.42
**Blood cancers**	1	1	0	0.98	1	14 899	12 397	16.79
**Calculus of kidney, ureter**	0.85	0.95	0.10	0.98	1	18 233	11 458	37.16
**Cataract**	1	0.95	0.05	0.98	0.95	73 884	53 814	27.16
**Cervical cancer**	0.8	0.95	0.15	1	0.86	1096	810	26.09
**Colon polyp**	1	1	0	0.87	1	39 470	13 995	64.54
**Disk disorders**	0.95	1	0.05	1	1	56 187	35 375	37.04
**Dysmenorrhea**	1	1	0	1	1	6689	2880	56.94
**Erectile dysfunction**	0.95	1	0.05	0.92	1	23 816	14 130	40.67
**Hepatitis, hepatocellular disease**	0.95	0.95	0	0.98	1	43 622	29 045	33.42
**Hyperthyroidism**	0.63	0.75	0.12	1	0.83	10 020	7043	29.71
**Interstitial cystitis**	0.8	0.95	0.15	1	1	1349	726	46.18
**Liver cancer**	0.85	0.9	0.05	1	0.95	8939	7227	19.15
**Osteoporosis**	0.75	0.75	0	1	0.95	52 317	37 582	28.16
**Other cancers**	1	0.9	0.10	0.98	1	53 319	42 463	20.36
**Other neurologic disorders**	1	1	0	0.98	1	11 869	6461	45.56
**Pancreatitis**	0.8	0.85	0.05	1	0.85	1791	1027	42.66
**Peptic ulcer**	1	0.95	0.05	0.96	0.96	9880	4170	57.79
**PMS, PMDD**	1	1	0	0.98	0.91	3045	1234	59.47
**RLS**	0.95	1	0.05	0.98	0.95	5269	3111	40.96
**Substance use disorders, including alcohol**	0.75	1	0.25	0.93	0.90	36 328	17 389	52.13

Abbreviations: ICD, International Classification of Diseases; NPV, negative predictive value; PMDD, premenstrual dysphoric disorder; PMS, Premenstrual syndrome; PPV, positive predictive value; RLS, Restless legs syndrome.

### PPV for ≥2 ICD codes

When requiring ≥2 ICD codes for case identification, the overall PPV showed mild improvement. The mean PPV across all conditions for ≥2 ICD codes was 0.94 (range 0.68-1.0). At the individual condition level, most had minimal PPV changes when requiring additional codes. Substance use disorders had the largest PPV increase with ≥2 ICD codes, although the PPV using ≥1 code still exceeded our acceptable threshold (Table 2).

### Conditions with low PPV

Some conditions demonstrated lower PPV due to common sources of miscoding. For hyperthyroidism, few had evidence of hypothyroidism or Hashimoto’s thyroiditis while others were actually diagnosed with transient or subacute forms of hyperthyroidism (eg, subacute thyroiditis, Hashimoto’s toxicosis). For osteoporosis and aortic aneurysm, misclassification frequently occurred when ICD codes were entered when diagnostic testing was ordered, before confirmation of the diagnosis. Additionally, both conditions were often coded when test results indicated borderline or at-risk findings (eg, osteopenia rather than true osteoporosis). For substance use disorders, false positives sometimes arose from drug screen results flagged out-of-context. For example, charts showed amphetamine-positive labs in patients appropriately prescribed ADHD medications, without any additional clinical evidence of misuse.

### NPV

The NPV was high across all conditions, with a mean of 0.98 (range 0.87 for colon polyp to 1.0 for 11 conditions; Table 2).

### Inter-rater agreement

For charts reviewed by both abstractors, the inter-rater agreement was excellent,^27^ with a mean kappa of 0.96 (range 0.83-1.0; Table 2).

### Sensitivity and specificity

Sensitivity and specificity were high (>0.80 and >0.85, respectively) for ≥1 ICD code case definitions for all conditions. Complete validation statistics, with corresponding 95% CI for all metrics, are shown in Appendix Table S3.

### Sample size tradeoff (% of cases lost)

**Figure 2:**
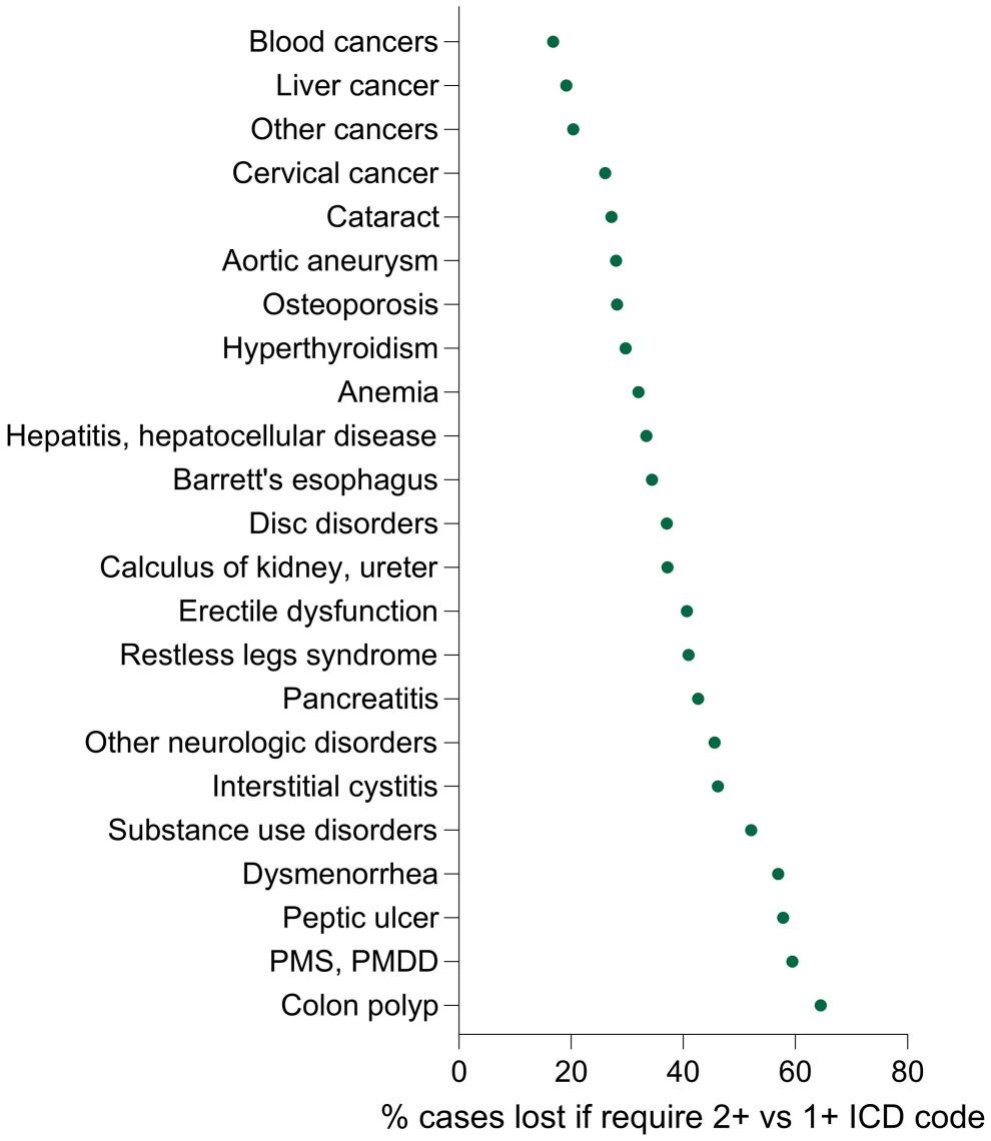
Sample size tradeoff (percent of cases lost) when using stricter ≥2 ICD codes case definition vs ≥1 ICD code case definition to define a condition case, for 23 MWI chronic conditions and condition groups assessed in chart review in the UCLA Health EHR, 2013-2019. ICD, International Classification of Diseases; PMDD, premenstrual dysphoric disorder; PMS, premenstrual syndrome.

When requiring ≥2 ICD codes to define cases (vs ≥1 code), the percent of cases lost ranged from 16.79% for blood cancers to 64.54% for colon polyp, with a mean sample size loss of 38.10% (Table 2, Figure 2). The four cancers had the lowest sample size tradeoffs, while colon polyp, peptic ulcer, and female gynecologic conditions had the highest.

### Condition coding frequency

The cancers were most likely to be coded many times for a patient, while colon polyp, peptic ulcer, and nonmalignant female gynecologic conditions were least likely. Blood cancer averaged 30.74 coding incidences per patient with at least one code, while colon polyp averaged 1.93 times per patient (Appendix Table S2).

## Discussion

We examined the accuracy of ICD-based case definitions for a wide range of chronic conditions without established algorithms in the EHR. For many chronic conditions, a single ICD code was sufficient to accurately identify cases when assessed over multiple years of EHR data, as in our 2013-2019 dataset. Additionally, while stricter case definitions (ie, requiring ≥2 codes) modestly improved PPV, they also reduced sample size, a tradeoff that varied in magnitude by condition.

Some conditions demonstrated lower PPVs, which may reflect limitations in ICD-based coding. First, some ICD codes lack sufficient granularity to distinguish between distinct clinical states. For example, there is no specific ICD code for osteopenia. Instead, there is a nonspecific^28^^,^^29^ code for “other disorders of bone density,” which may lead to selecting the closest available diagnosis, osteoporosis, even if the patient does not meet diagnostic criteria. Second, many EHRs including Epic require providers to assign a diagnosis when placing each order; however, no specific ICD codes exist to indicate that a condition is merely under evaluation. This may lead to premature or provisional coding of diagnoses before confirmation, as we observed in hyperthyroidism, osteoporosis, and aortic aneurysm. For these conditions with low PPV reflecting limitations in ICD codes, case definitions incorporating additional EHR domains may improve case ascertainment, although prior studies developing case definitions with ICD codes, medications, and procedures suggest that increased complexity does not always confer benefit.^10^^,^^30^^,^^31^

We also observed condition-specific coding frequency patterns aligned with prior literature. First, female gynecologic conditions are historically underrepresented in health records^32^ and in our dataset, were only infrequently coded multiple times. For example, although pooled prevalence estimates for PMS are 50%,^33^ our observed prevalence was merely 0.68%. This undercoding may reflect both fewer healthcare encounters among younger, reproductive-age women,^34^ and broader systemic undervaluation of women’s health, including perceptions of lower severity and lower reimbursement priority.^32^^,^^35^ Colon polyps, while reliably coded with a single code (likely following polypectomy), were also the least likely to be coded more than once or on many instances. This pattern likely reflects the fact that while polyp recurrence after polypectomy is common, recurrence rates still often fall below 50%, especially for shorter follow-up periods.^36^^,^^37^ In contrast, cancers were most likely to be coded not only more than once, but also most frequently, likely due to the perceived seriousness of diagnosis,^38–40^ involvement of multidisciplinary specialty care, and extensive diagnostic and therapeutic interventions. These patterns also illustrate how condition-specific coding frequency can inform development of realistic case definitions for different conditions.

While prior case definitions have been developed for many chronic conditions across administrative claims and EHR data,^3^^,^^4^^,^^9–12^^,^^22^^,^^41^ we conducted chart review validation for conditions that lack validated EHR-based case definitions. Our results are consistent with prior literature on EHR-based algorithms^9–12^: for some conditions, requiring ≥2 ICD codes improves accuracy, while others reach sufficient accuracy with a single ICD code, especially when assessed over multiple years of data. We also found that applying definitions developed in claims data (eg, CCW) may not always generalize to EHR settings. Our chart review included validation of four conditions/condition groups overlapping with CCW case definitions: cancers, osteoporosis, anemia, and cataract. While CCW definitions often required one inpatient or two outpatient codes, our EHR study demonstrated that one ICD code, regardless of outpatient or inpatient setting, provided sufficient validity for these four conditions and condition groups.

Our study has several strengths. The UCLA Health System serves a large and socioeconomically diverse population, to maximize generalizability with broad representation by age, race/ethnicity, insurance type,^42^ and social vulnerability. Including adults of all ages enabled us to assess chronic conditions affecting adults across the lifespan (ranging from dysmenorrhea to cataracts), an advantage over CCW’s Medicare-based definitions that omit diseases skewed toward younger and middle-aged adults. Additionally, with multiple insurance types, we could account for varying patterns of healthcare utilization and reimbursement policies.^43^ In total, we performed chart review on a wide range of chronic conditions affecting all organ systems managed in both inpatient and outpatient settings, and inclusive of age- and sex-specific conditions. This rigorous, comprehensive chart review was conducted on thousands of charts and condition combinations and encompassed the entire chart, including primary and specialty care, historic notes, and outside records when available. Clinical notes are especially advantageous where objective data are missing. For transfer patients, we confirmed prior cancer diagnoses through detailed primary care physician (PCP) documentation when pathology records were unavailable. Finally, by quantifying tradeoffs of stricter ICD-based case definitions, our findings help guide researchers to balance accuracy and sample size based on study objectives.

Our results must be interpreted within the following considerations. First, results are influenced by the length and completeness of EHR data, including availability of historic records. Chronic conditions may not be discussed, documented, or billed at each visit and thus may not appear as a case, particularly in cross-sectional analyses. Longer EHR histories help mitigate under-ascertainment, particularly for conditions infrequently coded. UCLA Health adopted Epic in 2013,^19^ but historic records from the 1990s were variably available. Second, a related consideration is availability of linked outside records, including historical notes and reports from other health systems. UCLA EHR regularly includes records from other local hospital systems. Finally, characteristics of the chart review cohort used can affect the observed validity. Patients with greater continuity of care may have more encounters and diagnostic studies, increasing the likelihood of coded diagnoses, albeit not necessarily more accurate coding.

Other limitations concern the interpretability of validation statistics. First, we did not assess the coding accuracy of *exactly one* ICD code, as our analysis focused on cohorts defined by ≥1 and ≥2 ICD codes. However, from a research standpoint, it is often more useful to understand the minimum number of codes needed to define a valid cohort than to isolate cases with only a single instance of coding. Thus, we validated both ≥1 and ≥2 ICD code case definitions, recognizing that there is overlap between these patient cohorts (ie, patients in the ≥2 code cohort are also in the ≥1 code cohort). By defining a potential case population as individuals with ≥1 and ≥2 ICD code anytime in our 7-year study period, we could examine validity of case definitions that would minimize sample size loss, unlike prior studies that required codes within a restricted 1- or 2-year interval.^4^^,^^44^ Second, we recognize the limited utility of NPV when randomly sampling those without relevant ICD codes, especially for conditions of lower prevalence. However, given our study of 23 diverse chronic conditions, it was not feasible to generate a targeted controls cohort for each condition.

Finally, our study was conducted at a single academic health system. Institutional factors, such as the use of professional coders, teaching hospital status, and local billing practices, can influence coding behavior. Additionally, differences may likely be seen in nonacademic institutions and community clinics serving distinct populations (eg, federally qualified health centers) and without a focus on medical training. However, although UCLA is a single institution, it operates an expansive health network that spans over 200 hospitals and clinics across Southern California (Appendix Figure S1)^45^ and serves diverse communities, supporting broad generalizability.

For EHR-based chronic condition case ascertainment, a single ICD code may provide sufficient accuracy for many conditions, particularly when assessed over multiple years. However, given the wide variability in PPV in our study, case definitions should be assessed on a condition-by-condition basis. Case definitions that perform well for one condition neither generalize to others nor across different types of data (eg, claims vs EHR). Ultimately, the choice of case definition should reflect the study objectives. Index disease studies require a cohort with minimal false positives (ie, a case definition that maximizes PPV and specificity), while multimorbidity and comorbidity measurement may prioritize broader condition capture across all conditions, requiring particular attention to sensitivity. As EHR data become increasingly central to research and clinical decision-making, rigorous evidence-based approaches to case ascertainment are critical to ensure validity, generalizability, and impact.

## Supplementary material

Supplementary material is available at *The Journals of Gerontology, Series A: Biological Sciences and Medical Sciences* online.

## Funding

This work was supported by the National Institute on Aging at the National Institutes of Health (5R01AG083370 to M.Y.W.), which was not involved in any aspect of study design, data collection and analysis, interpretation of results, or decisions relating to the final manuscript.

## Supplementary Material

glag157_Supplementary_Data

## Data Availability

UCLA EHR data is available for researchers through the UCLA Clinical & Translational Science Institute (CTSI) following approval of request with adequate scientific justification for research project.
