# Supplementary material for: Validating ICD code case definitions for condition case ascertainment in multimorbidity measurement: a retrospective chart review
Source: J Gerontol A Biol Sci Med Sci. 2026 Jun 10;81(8):glag157. doi: 10.1093/gerona/glag157 (PMC13372667; doi:10.1093/gerona/glag157)
Supplement: glag157_Supplementary_Data [file glag157_supplementary_data.docx]

**Supplemental Material**

Validating ICD code case definitions for condition case ascertainment in multimorbidity measurement: a retrospective chart review

Ashley J. Kang, BS^a^, Chi-Hong Tseng, PhD^a^, Melissa Y. Wei, MD, MPH, MS^a,b^

^a^ Division of General Internal Medicine and Health Services Research, Department of Medicine, University of California, Los Angeles, 1100 Glendon Ave., Suite 900, Los Angeles, CA 90024, USA

^b^ Center for the Study of Healthcare Innovation, Implementation and Policy, VA Greater Los Angeles Healthcare System, Los Angeles, CA, USA

**Table of Contents Page**

| **Title Page** | 1 |
| --- | --- |
| **Appendix Table 1.**  Full list of conditions in the multimorbidity-weighted index (MWI) and whether a validated case definition existed for electronic health record (EHR) data. For the 23 conditions and condition groups in MWI without existing case definitions, we performed chart review in the UCLA Health EHR, 2013-2019. | 2 |
| **Appendix Table 2.** Total number coded diagnoses and average frequency of condition coding for 23 MWI chronic conditions and condition groups assessed in UCLA Health EHR chart review, 2013-2019. | 7 |
| **Appendix Table 3.** Full validation statistics for 23 conditions and condition groups chart reviewed in the UCLA EHR. | 8 |
| **Appendix Figure 1.** UCLA Health clinic and hospital locations represented by red pin drops, from which the study sample was drawn, 2013-2019. | 10 |
| **Appendix File 1.** Retrospective medical chart review protocol for identifying chronic conditions from the multimorbidity-weighted index (MWI) in the EHR. | 11 |
|  |  |

**Appendix Table 1.**  Full list of conditions in the multimorbidity-weighted index (MWI) and whether a validated case definition existed for electronic health record (EHR) data. For the 23 conditions and condition groups in MWI without existing case definitions, we performed chart review in the UCLA Health EHR, 2013-2019.

| **Condition or condition group** | **No validated case definition exists in the EHR, assessed in chart review** | **Validated case definition exists in the EHR, did not chart review** |
| --- | --- | --- |
| ***Cardiovascular*** |  |  |
| Aortic aneurysm (aortic aneurysm and dissection) | X |  |
| Angina |  | X |
| Arrhythmias (including atrial fibrillation, atrial flutter) |  | X |
| Automated implantable cardioverter  defibrillator (AICD) |  | X |
| Congestive heart failure, Cardiomyopathy |  | X |
| Coronary artery bypass graft (CABG) surgery |  | X |
| Coronary artery disease |  | X |
| High blood pressure, hypertension |  | X |
| Myocardial infarction |  | X |
| Peripheral artery disease, Atherosclerosis of extremities |  | X |
| Valvular heart disease (Aortic insufficiency, Aortic valve disease, Aortic valve replacement, Aortic valve stenosis, Aortic and mitral valve disorder, Mitral valve prolapse, Mitral regurgitation, Mitral stenosis, Mitral valve disease, Mitral valve replacement, Pulmonary valve disease, Tricuspid valve disease, Valve implant) |  | X |
| ***Endocrine*** |  |  |
| Diabetes mellitus |  | X |
| Elevated cholesterol, Hyperlipidemia |  | X |
| Hyperthyroidism | X |  |
| Hypothyroidism |  | X |
| Thyroid nodule, goiter |  | X* |
| ***Gastrointestinal*** |  |  |
| Barrett’s esophagus | X |  |
| Cirrhosis, Liver failure, Liver transplant |  | X |
| Colon polyp | X |  |
| Diverticulosis, Diverticulitis |  | X |
| Gallstones |  | X |
| Hepatitis, hepatocellular disease | X |  |
| Inflammatory bowel disease  (Crohn’s disease, Ulcerative colitis) |  | X |
| Pancreatitis | X |  |
| Ulcer, peptic | X |  |
| ***Hematologic*** |  |  |
| Anemia (excluding acute post-hemorrhagic anemia) | X |  |
| Venous thromboembolism  (Pulmonary embolism, Deep vein thrombosis) |  | X |
| ***Immunologic*** |  |  |
| Acquired immune deficiency syndrome (AIDS) |  | X |
| ***Integumentary*** |  |  |
| Solar actinic keratosis |  | X* |
| ***Musculoskeletal*** |  |  |
| Connective tissue disease  (Sjogren’s disease, Dermatomyositis, Polymyositis, Systemic lupus erythematosus) |  | X |
| Disc disorders | X |  |
| Gout |  | X |
| Hip fracture |  | X |
| Hip replacement surgery |  | X |
| Knee replacement surgery |  | X |
| Osteoarthritis |  | X |
| Osteoporosis | X |  |
| Rheumatoid arthritis |  | X |
| Vertebral fracture |  | X |
| Wrist fracture |  | X* |
| ***Nervous*** |  |  |
| Amyotrophic lateral sclerosis (ALS), Motor neuron disease |  | X |
| Cerebrovascular disease, Stroke |  | X |
| Dementia, Alzheimer disease |  | X |
| Migraine headache |  | X |
| Multiple sclerosis |  | X |
| Other neurologic disorders (those without their own MWI category/unspecified elsewhere) | X |  |
| Paralytic syndrome |  | X |
| Parkinson disease |  | X |
| Restless legs syndrome | X |  |
| Seizure disorder, Epilepsy |  | X |
| Transient ischemic attack |  | X |
| ***Oncologic*** |  |  |
| Basal cell carcinoma |  | X* |
| Bladder cancer |  | X |
| Blood cancers (Leukemia,  Lymphoma, Multiple myeloma) | X |  |
| Breast cancer |  | X |
| Cervical cancer | X |  |
| Colorectal cancer |  | X |
| Lung cancer |  | X |
| Liver cancer | X |  |
| Melanoma |  | X* |
| Other cancers (those without their own MWI category/unspecified elsewhere), Neoplasms of unspecified nature | X |  |
| Ovarian cancer |  | X |
| Prostate cancer |  | X |
| Squamous cell carcinoma |  | X* |
| Uterine cancer |  | X |
| ***Ophthalmologic*** |  |  |
| Cataract | X |  |
| Glaucoma |  | X |
| Macular degeneration |  | X |
| ***Psychiatric*** |  |  |
| Anxiety |  | X |
| Depression and related psychiatric conditions |  | X |
| Substance use disorders, including alcohol | X |  |
| ***Pulmonary*** |  |  |
| Asthma |  | X |
| Chronic pulmonary diseases (including COPD, interstitial lung disease) |  | X |
| ***Renal*** |  |  |
| Calculus of kidney and ureter | X |  |
| Chronic kidney disease (CKD), other chronic renal diseases |  | X |
| Interstitial cystitis | X |  |
| ***Reproductive*** |  |  |
| Benign breast disease |  | X* |
| Benign prostatic hyperplasia (BPH) |  | X* |
| Ectopic and molar pregnancy |  | X* |
| Endometriosis |  | X |
| Erectile dysfunction | X |  |
| Dysmenorrhea | X |  |
| Polycystic ovary syndrome (PCOS) |  | X |
| Premenstrual syndrome (PMS), Premenstrual dysphoric disorder (PMDD) | X |  |
| Prostate surgery for benign prostatic hyperplasia |  | X* |
| Uterine fibroid(s), Benign uterine growth, Leiomyoma |  | X |

*****MWI conditions are weighted to their impact on the Short Form-36 physical functioning scale and these ten noted conditions have a weight of 0 (no impact on physical functioning). As such, they are excluded from this analysis and were not considered for chart review.

**Appendix Table 2.** Total number coded diagnoses and average frequency of condition coding for 23 MWI chronic conditions and condition groups assessed in UCLA Health EHR chart review, 2013-2019.

| **Condition** | **Total number of coded diagnoses** | **Number of unique patients with ≥1 ICD code** | **Number of unique patients with ≥2 ICD codes** | **Average number of times person coded with condition** |
| --- | --- | --- | --- | --- |
| Anemia | 570194 | 89257 | 60678 | 6.39 |
| Aortic aneurysm (aortic aneurysm and dissection) | 44734 | 6504 | 4684 | 6.88 |
| Barrett's esophagus | 13422 | 3065 | 2010 | 4.38 |
| Blood cancers | 457993 | 14899 | 12397 | 30.74 |
| Calculus of kidney, ureter | 85110 | 18233 | 11458 | 4.67 |
| Cataract | 385330 | 73884 | 53814 | 5.22 |
| Cervical cancer | 13989 | 1096 | 810 | 12.76 |
| Colon polyp | 76317 | 39470 | 13995 | 1.93 |
| Disc disorders | 249254 | 56187 | 35375 | 4.44 |
| Dysmenorrhea | 14018 | 6689 | 2880 | 2.10 |
| Erectile dysfunction | 72737 | 23816 | 14130 | 3.05 |
| Hepatitis, hepatocellular disease | 316752 | 43622 | 29045 | 7.26 |
| Hyperthyroidism | 76585 | 10020 | 7043 | 7.64 |
| Interstitial cystitis | 5571 | 1349 | 726 | 4.13 |
| Liver cancer | 193738 | 8939 | 7227 | 21.67 |
| Osteoporosis | 338833 | 52317 | 37582 | 6.48 |
| Other cancers | 914643 | 53319 | 42463 | 17.15 |
| Other neurologic disorders | 49966 | 11869 | 6461 | 4.21 |
| Pancreatitis | 8280 | 1791 | 1027 | 4.62 |
| Peptic ulcer | 23516 | 9880 | 4170 | 2.38 |
| Premenstrual syndrome (PMS), Premenstrual dysphoric disorder (PMDD) | 6772 | 3045 | 1234 | 2.22 |
| Restless legs syndrome (RLS) | 19997 | 5269 | 3111 | 3.80 |
| Substance use disorders, including alcohol | 136133 | 36328 | 17389 | 3.75 |

**Abbreviations.** ICD: International Classification of Diseases

**Appendix Table 3.** Full validation statistics* for 23 conditions and condition groups chart reviewed in the UCLA EHR.

| **Condition** | **PPV ≥1 (95% CI)** | **PPV ≥2 (95% CI)** | **NPV (95% CI)** | **Cohen’s kappa (95% CI)** | **Specificity ≥1 (95% CI)** | **Sensitivity ≥1 (95% CI)** |
| --- | --- | --- | --- | --- | --- | --- |
| Anemia | 0.900 (0.683, 0.988) | 1.000 (0.832, 1.000) | 1.000 (0.900, 1.000) | 1.000 (1.000, 1.000) | 0.946 (0.818, 0.993) | 1.000 (0.815, 1.000) |
| Aortic aneurysm (aortic aneurysm and dissection) | 0.789 (0.544, 0.940) | 0.684 (0.435, 0.874) | 1.000 (0.928, 1.000) | 1.000 (1.000, 1.000) | 0.925 (0.818, 0.979) | 1.000 (0.782, 1.000) |
| Barrett's esophagus | 0.950 (0.751, 0.999) | 1.000 (0.832, 1.000) | 1.000 (0.925, 1.000) | 0.908 (0.783, 1.000) | 0.979 (0.889, 1.000) | 1.000 (0.824, 1.000) |
| Blood cancers | 1.000 (0.832, 1.000) | 1.000 (0.832, 1.000) | 0.978 (0.885, 1.000) | 1.000 (1.000, 1.000) | 1.000 (0.921, 1.000) | 0.952 (0.762, 0.999) |
| Calculus of kidney, ureter | 0.850 (0.621, 0.968) | 0.950 (0.751, 0.999) | 0.975 (0.868, 0.999) | 1.000 (1.000, 1.000) | 0.929 (0.805. 0.985) | 0.944 (0.727, 0.999) |
| Cataract | 1.000 (0.832, 1.000) | 0.950 (0.751, 0.999) | 0.976 (0.874, 0.999) | 0.952 (0.860, 1.000) | 1.000 (0.914, 1.000) | 0.952 (0.762, 0.999) |
| Cervical cancer | 0.800 (0.563, 0.943) | 0.950 (0.751, 0.999) | 1.000 (0.926, 1.000) | 0.862 (0.713, 1.000) | 0.923 (0.815, 0.979) | 1.000 (0.794, 1.000) |
| Colon polyp | 1.000 (0.832, 1.000) | 1.000 (0.832, 1.000) | 0.868 (0.719, 0.956) | 1.000 (1.000, 1.000) | 1.000 (0.894, 1.000) | 0.800 (0.593, 0.932) |
| Disc disorders | 0.950 (0.751, 0.999) | 1.000 (0.832, 1.000) | 1.000 (0.912, 1.000) | 1.000 (1.000, 1.000) | 0.976 (0.871, 0.999) | 1.000 (0.824, 1.000) |
| Dysmenorrhea | 1.000 (0.832, 1.000) | 1.000 (0.832, 1.000) | 1.000 (0.920, 1.000) | 1.000 (1.000, 1.000) | 1.000 (0.920, 1.000) | 1.000 (0.832, 1.000) |
| Erectile dysfunction | 0.950 (0.751, 0.999) | 1.000 (0.832, 1.000) | 0.919 (0.781, 0.983) | 1.000 (1.000, 1.000) | 0.971 (0.851, 0.999) | 0.864 (0.651, 0.971) |
| Hepatitis, hepatocellular disease | 0.950 (0.751, 0.999) | 0.950 (0.751, 0.999) | 0.976 (0.874, 0.999) | 1.000 (1.000, 1.000) | 0.976 (0.874, 0.999) | 0.950 (0.751, 0.999) |
| Hyperthyroidism | 0.632 (0.384, 0.837) | 0.750 (0.509, 0.913) | 1.000 (0.926, 1.000) | 0.832 (0.650, 1.000) | 0.873 (0.755, 0.947) | 1.000 (0.735, 1.000) |
| Interstitial cystitis | 0.800 (0.563, 0.943) | 0.950 (0.751, 0.999) | 1.000 (0.926, 1.000) | 1.000 (1.000, 1.000) | 0.923 (0.815, 0.979) | 1.000 (0.794, 1.000) |
| Liver cancer | 0.850 (0.621, 0.968) | 0.900 (0.683, 0.988) | 1.000 (0.926, 1.000) | 0.954 (0.865, 1.000) | 0.941 (0.838, 0.988) | 1.000 (0.805, 1.000) |
| Osteoporosis | 0.750 (0.509, 0.913) | 0.750 (0.509, 0.913) | 1.000 (0.912, 1.000) | 0.949 (0.849, 1.000) | 0.889 (0.760, 0.963) | 1.000 (0.782, 1.000) |
| Other cancers | 1.000 (0.832, 1.000) | 0.900 (0.683, 0.988) | 0.976 (0.871, 0.999) | 1.000 (1.000, 1.000) | 1.000 (0.912, 1.000) | 0.952 (0.762, 0.999) |
| Other neurologic disorders | 1.000 (0.832, 1.000) | 1.000 (0.832, 1.000) | 0.976 (0.871, 0.999) | 1.000 (1.000, 1.000) | 1.000 (0.912, 1.000) | 0.952 (0.762, 0.999) |
| Pancreatitis | 0.800 (0.563, 0.943) | 0.850 (0.621, 0.968) | 1.000 (0.923, 1.000) | 0.853 (0.692, 1.000) | 0.920 (0.808, 0.978) | 1.000 (0.794, 1.000) |
| Peptic ulcer | 1.000 (0.832, 1.000) | 0.950 (0.751, 0.999) | 0.957 (0.855, 0.995) | 0.955 (0.869, 1.000) | 1.000 (0.921, 1.000) | 0.909 (0.708, 0.989) |
| Premenstrual syndrome (PMS), premenstrual dysphoric disorder (PMDD) | 1.000 (0.832, 1.000) | 1.000 (0.832, 1.000) | 0.979 (0.889, 1.000) | 0.908 (0.783, 1.000) | 1.000 (0.925, 1.000) | 0.952 (0.762, 0.999) |
| Restless legs syndrome (RLS) | 0.950 (0.751, 0.999) | 1.000 (0.832, 1.000) | 0.978 (0.885, 1.000) | 0.953 (0.861, 1.000) | 0.978 (0.885, 1.000) | 0.950 (0.751, 0.999) |
| Substance use disorders, including alcohol | 0.750 (0.509, 0.913) | 1.000 (0.832, 1.000) | 0.927 (0.801, 0.985) | 0.898 (0.761, 1.000) | 0.884 (0.749, 0.961) | 0.833 (0.586, 0.964) |

*****We used the binomial confidence interval calculator (<https://statpages.info/confint.html#Binomial>) for all validation statistics except kappa. For kappa, we calculated the confidence interval using: McHugh ML. Interrater reliability: the kappa statistic. *Biochem Med (Zagreb)*. 2012;22(3):276-82. PMID: 23092060; PMCID: PMC3900052.

**Appendix Figure 1.** UCLA Health clinic and hospital locations represented by red pin drops,* from which the study sample was drawn, 2013-2019.


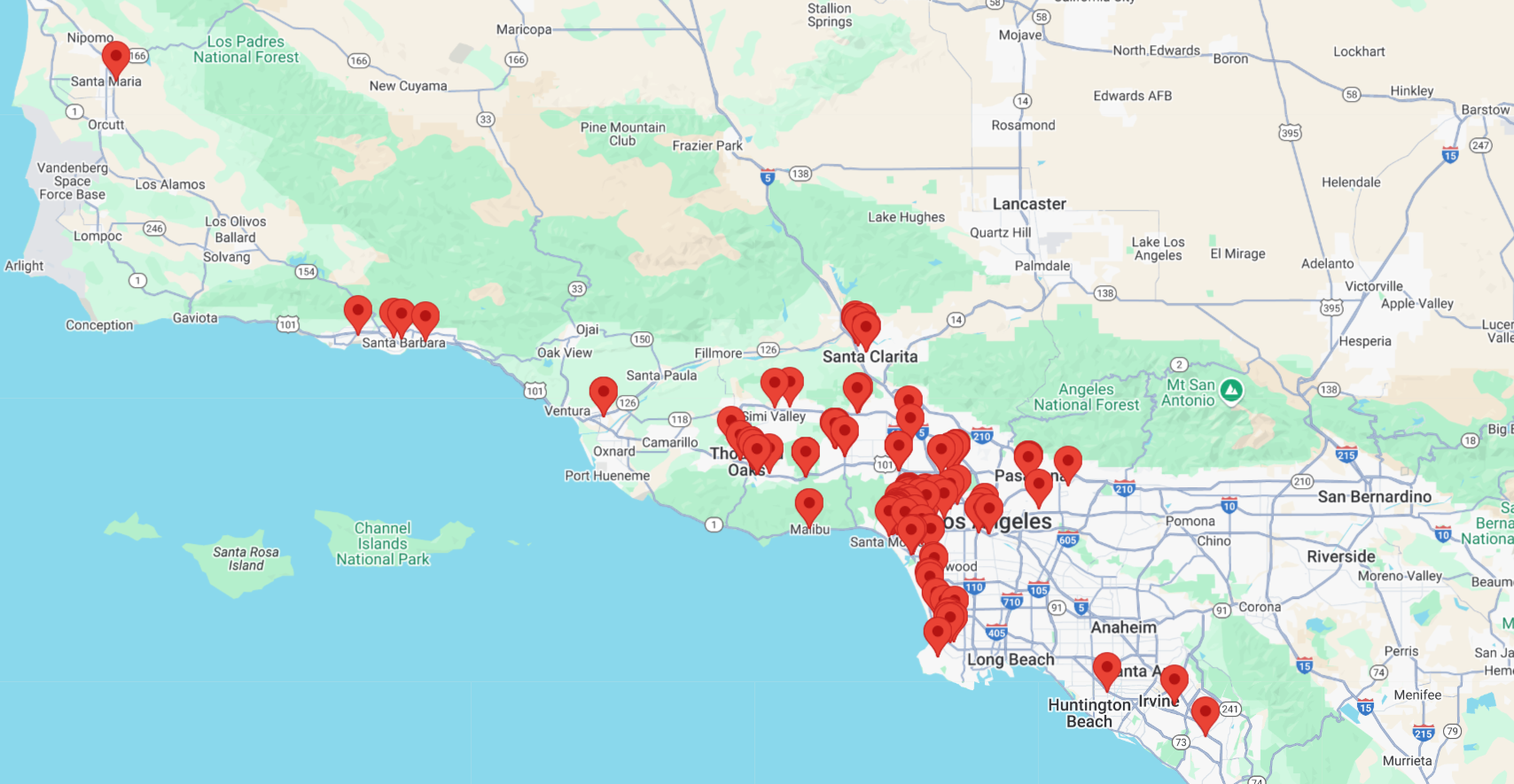


*Image taken from UCLA Health website “Finding a UCLA Health location.” <https://www.uclahealth.org/locations>

**Appendix File 1.** Retrospective chart review protocol for identifying chronic conditions from the multimorbidity-weighted index (MWI) in the EHR.

## **______________________________________________________________________________**

**Purpose**:

This medical chart review was conducted to identify the presence (**case positive**) or absence (**case negative**) of chronic conditions among patients sampled for retrospective chart review. Conditions assessed are those included in the multimorbidity-weighted index (MWI) that lacked previously validated EHR-based case definitions.

## **______________________________________________________________________________**

## **General Chart Review Protocol**

These guidelines and definitions apply to all individual condition protocols and are not repeated in each condition’s instructions for brevity:

- **MWI definition of chronic condition**: refers to conditions with a minimum duration of 3 months, considered largely irreversible and persistent through adulthood. These include chronic diseases, long-term sequelae of serious illness (e.g. stroke with residual deficits), surgical procedures indicating severe disease progression that failed conservative management (e.g., knee replacement), and conditions that require some degree of ongoing management via medications, behavioral and lifestyle modification, and/or routine surveillance.
- **Case positive**: any evidence of the condition (per protocol), including documented history of the condition in the EHR, at any time.
- **Case negative**: The entire chart must be reviewed before assigning a “case negative.”
- **Note review:** notes can either serve as (1) a confirmation of another data domain (e.g. meds, labs), or (2) if used on their own, must include detailed documentation of the condition to be considered case positive. For relevant notes (primary care physician (PCP) and applicable specialists), consider all types of notes (e.g. progress notes, history & physical (H&P), consults, procedures, operative notes) except for non-encounter telephone notes. Also review any notes where relevance isn’t immediately apparent (e.g. notes not labeled with specific provider).
- **Definitions for notes review:**
  - ***One mention in note*:** a brief mention of condition anywhere in the note (e.g., problem list, past medical history, subjective history, assessment, plan), without additional detail necessary.
  - ***One detailed note*:** includes explicit mention of condition and further supporting details, anywhere in the note. Details include:
    - Diagnostic evidence (e.g., labs, imaging, pathology, staging, measurements)
    - Treatment plans (e.g., medications, referrals, procedures, other treatments, counseling on lifestyle management)
    - Detailed history of condition (e.g., dates, course, complications, status of condition (e.g. stable, high priority, de-prioritized)
  - **Providers considered PCPs**: internal medicine, geriatrics, family medicine, primary care, obstetrics and gynecology (OB/GYN), pediatrics, and UCLA-specific primary care (e.g., East/West Medicine).
  - **Additional note types included:**
    - **Discharge summary:** inpatient discharge documentation.
    - **Admission History & Physical (H&P):** initial evaluation at hospital admission.
    - **Emergency Department (ED) provider notes**: emergency department visit documentation.
- **Supplemental sources**:
  - **Pathology, imaging, labs**: Include all relevant reports that might reference the condition. Also review all pathology, imaging, lab reports, and notes even if the labeling is nonspecific (e.g., “outside pathology).
    - Example: for Barrett’s esophagus, include pathology reports for “esophagus/GI” as well as generic reports (e.g., “pathology finding” or “outside pathology”) but not clearly unrelated reports (e.g., “cervical cytology”).
  - **Labs:** interpret using UCLA EHR-specific reference ranges and normal/abnormal flags.
  - **Medication list:** search all past and active medications. A medication mentioned in a note must be explicitly linked to the condition within the same note.
- **Conducting chart review**: review each component (medication list, labs, notes, imaging, pathology, etc) as applicable in chronological order.
- **Search strategy**: In UCLA Care Connect, there is a “diagnosis” filter to select notes with relevant ICD codes/condition names. Used this to start with notes most likely to contain relevant information; however, still reviewed all notes relevant to the condition according to protocols below.

**Individual chronic condition protocols for chart review**

We assessed 23 conditions from the multimorbidity-weighted index (MWI) that lacked validated EHR-based case definitions, based on literature review. The 23 chronic conditions assessed include: anemia; aortic aneurysm; Barrett’s esophagus; blood cancers; calculus of kidney, ureter; cataract; cervical cancer; colon polyp; disc disorders; dysmenorrhea; erectile dysfunction; hepatitis, hepatocellular disease; hyperthyroidism; interstitial cystitis; liver cancer; osteoporosis; other cancers; other neurologic disorders; pancreatitis; peptic ulcer; premenstrual syndrome (PMS), premenstrual dysphoric disorder (PMDD); restless legs syndrome (RLS); substance use disorders.

The remaining MWI conditions already had validated EHR-based definitions and were not included in the present chart review. **Appendix Table 1** provides the full list of MWI conditions and indicates which were assessed in this study.

**ANEMIA (EXCLUDING ACUTE POST-HEMORRHEGIC ANEMIA)**

1. Check lab results for hemoglobin, typically reported within CBC panels (e.g., CBC, CBC with automated differential).

*Note: Make sure person has abnormal values that are low.*

- 1. Two occurrences of abnormal low hemoglobin values with test dates at least 90 days apart 🡪 case positive.
  2. One occurrence of low hemoglobin value anytime, or two occurrences of low hemoglobin values less than 90 days apart 🡪 search notes. These lab results plus 1 mention of anemia in physician note* 🡪 case positive.
  3. Normal hemoglobin lab results or no labs present 🡪 search notes. 1 detailed physician note* 🡪 case positive.

*Note: Our scope of anemia covers all types of anemia except when specified as acute posthemorrhagic anemia (anemia due to acute blood loss, which can happen with specific events e.g., trauma, surgeries, acute GI bleed).*

*Physician notes: Consider notes from PCP, hematology-oncology, and gastroenterology. If no case positive yet, search in discharge summary notes, admission notes, then ED provider notes. If at end of notes search do not find evidence for case positive, assign case negative.

**AORTIC ANEURYSM**

1. Check radiology imaging.
   1. For **abdominal aortic aneurysm (AAA),** search:
      1. ultrasound abdomen screening for AAA
      2. Incidental findings on CT abdomen, MRI abdomen, U/S abdomen ordered for other diagnostic studies
   - Abdominal aorta ≥ 3 cm on any of these imaging modalities 🡪 case positive
   - Or if imaging report mentions prior surgical repair of aneurysm: Surgical treatment typically indicated with AAA $\geq$5.5cm, growth $\geq$0.5cm/yr, or symptomatic AAA 🡪 case positive.
   1. For **thoracic aortic aneurysm,** search:
      1. Echocardiography: transthoracic echocardiogram (TTE) or transesophageal echocardiogram (TEE)
      2. Incidental findings on CT chest, MRI chest ordered for other diagnostic studies.
   - Thoracic aorta ≥ 3.5 cm on any of these imaging modalities 🡪 case positive
   - Or if imaging mentions evidence of surgical repair of aneurysm: Treatment indicated with aortic diameter >5.5 cm (4-5 cm for Marfan syndrome**), aortic diameter >4.5 cm and undergoing other heart surgery, rapid growth > 0.5 cm/year 🡪 case positive.
2. If no evidence of aneurysm on imaging or no imaging results, check physician notes*. 1 detailed physician note* 🡪 case positive.

*Physician notes: Consider notes from vascular surgery, cardiology and PCP. If no case positive yet, search in discharge summary notes, admission notes, then ED provider notes. If no evidence is found after reviewing ED notes, assign case negative.

** If using criteria based on Marfan syndrome size cutoff, must confirm Marfan syndrome in problem list or past medical history.

**BARRETT’S ESOPHAGUS**

1. Check pathology (reports often labelled esophagus, EGD, surgical GI, surgical pathology). Clinical impression of Barrett’s* on a pathology report (with or without mention of dysplasia) 🡪 case positive.

*On pathology reports, Barrett’s esophagus is not terminology often used. If “intestinal metaplasia” present in esophagus or gastroesophageal junction (GEJ) region 🡪 case positive.

1. If there are no pathology reports or no mention of Barrett’s esophagus in these reports, search for evidence of treatment in medication list (medication below).
   1. If treatment exists, treatment plus 1 mention in physician note* 🡪 case positive.
   2. If no evidence of treatment/medication exists, 1 detailed physician note* 🡪 case positive.

*Physician notes: Consider notes from gastroenterology and PCP. If no case positive yet, search for discharge summary notes, admission notes, then ED provider notes.

1. If neither 2a nor 2b is met once finish notes search, check for any relevant scanned-in outside records that were faxed in. If Barrett’s reported through an outside pathology report, then case positive. If not, 🡪 case negative.

**Medications: Proton pump inhibitors (PPIs)**

- omeprazole (Prilosec, Zegerid)
- lansoprazole (Prevacid)
- pantoprazole (Protonix)
- rabeprazole (AcipHex)
- esomeprazole (Nexium)
- dexlansoprazole (Dexilant)

**BLOOD CANCERS (LEUKEMIA, LYMPHOMA, MULTIPLE MYELOMA)**

1. Start with physician notes: 1 detailed physician* note 🡪 case positive.

**Terminology for blood cancers:**

- Lymphosarcoma
- Reticulosarcoma
- Malignant tumor/neoplasm of lymphatic/lymphoid, histiocytic tissue
- Lymphoma
  - Hodgkin lymphoma/Hodgkin’s disease
    - Hodgkin’s paragranuloma
    - Hodgkin’s granuloma
    - Hodgkin’s sarcoma
  - Non-Hodgkin lymphoma (NHL)
    - B-cell lymphoma
    - Small lymphocytic lymphoma (SLL)
  - Follicular lymphoma
  - Non-follicular lymphoma
    - Small cell B-cell lymphoma
    - Mantle cell lymphoma
    - Diffuse large B-cell lymphoma (DLBCL)
    - Lymphoblastic (diffuse) lymphoma
    - Burkitt tumor/Burkitt lymphoma
  - Mycosis fungoides
  - Sezary disease
  - Marginal zone lymphoma
  - Primary central nervous system (CNS) lymphoma
  - Anaplastic large cell lymphoma
  - Large cell lymphoma
  - Nodular lymphoma
  - T-cell lymphoma
  - Other mature T/NK-cell lymphomas
  - Malignant lymphomas
  - Other lymphomas
- Leukemia
  - Acute lymphoid/lymphocytic leukemia (ALL)
  - Chronic lymphoid/lymphocytic leukemia (CLL)
  - Subacute lymphoid/lymphocytic leukemia
  - Lymphoid/lymphocytic leukemia
  - Acute myeloid/myeloblastic leukemia (AML)
  - Chronic myeloid/myeloblastic leukemia (CML)
  - Subacute myeloid leukemia
  - Myeloid sarcoma
  - Myeloid leukemia
  - Acute monocytic/monoblastic leukemia
  - Chronic monocytic leukemia, chronic myelomonocytic leukemia (CMML)
  - Subacute monocytic leukemia
  - Monocytic leukemia
  - Acute erythremia and erythroleukemia
  - Chronic erythremia
  - Megakaryocytic leukemia
  - Mast cell leukemia
  - Other leukemia
- Multiple myeloma and malignant plasma cell neoplasms
  - Multiple myeloma
  - Plasma cell leukemia
  - Plasmacytoma
- Neoplasm of uncertain behavior of other lymphatic/lymphoid and hematopoietic tissue:
  - Myelodysplastic syndrome lesions
  - Myelodysplastic syndrome (MDS)
  - Others
- Malignant histiocytosis
- Leukemic reticuloendotheliosis
- Letterer-Siwe disease
- Malignant mast cell tumors
- Other immunoproliferative neoplasms
- Malignant immunoproliferative diseases
- Macroglobulinemia

*Note: Also keep in mind acronyms*

*Physician notes: Consider notes from hematology-oncology, surgical oncology, and PCP. If no case positive yet, search in discharge summary notes, admission notes, then ED provider notes. If at end of notes search do not find evidence for case positive, assign case negative.

**CALCULUS OF KIDNEY, URETER (KIDNEY, URETER STONES)**

1. Search in imaging (CT, ultrasound/US, or MRI/MR of abdomen, pelvis, urogram, kidneys, KUB (kidneys, ureters, bladder). If mention of calculus of kidney, ureter* 🡪 case positive

*Applicable terminology for calculus: stone, calculus, calculi, nephrolithiasis, ureterolithiasis, urolithiasis. These can be in kidney, ureter, urinary, urinary tract including the bladder, urethra/urethral, urinary tract, renal, as well as uric acid stones. Do not consider cholelithiasis (gallstones) for this condition.

1. If no case positive found in imaging or no imaging results, search lab results. Search for “stone analysis.” If stone analysis done (confirm in the “specimen information” section that the relevant type of stone is being assessed), then case positive.
2. If no stone analysis done, check for abnormal labs or relevant findings in medication list. Either of these can be used in combination with a mention in the notes to determine case positive.

**Labs**: check urinalysis, urine/urinalysis dipstick labs. Look for high red blood cell/“RBC” counts and “blood/urine blood” tests for blood in the urine (will be noted by values “1+ to 4+” (normal reference = 0), or terms suggesting blood in urine relative to normal reference of “negative”). Abnormal readings on any/either of these tests is considered as abnormal lab.

**Medications**: Check for presence of relevant findings in medication list (see medications list below).

- - - Alpha blockers (doxazosin, prazosin, terazosin)
    - Calcium channel blockers (amlodipine, nifedipine, diltiazem, nicardipine, felodipine)
    - Phosphodiesterase-5-inhibitors (PDE5): Sildenafil (Viagra, Revatio, Liqrev), vardenafil (Levitra, Staxyn), tadalafil (Cialis, Tadliq), avanafil (Stendra)
    - Corticosteroids (prednisone)

1. If find either abnormal lab or presence of relevant medication, then that plus 1 mention in physician note* 🡪 case positive
2. If no evidence of abnormal lab or medication exists, 1 detailed physician note* 🡪 case positive.

*Physician notes: Consider notes from urology and PCP. If no case positive yet, search in discharge summary notes, admission notes, then ED provider notes. If complete notes search and do not find evidence for case positive, assign case negative.

**CATARACT**

1. Start with physician note*. 1 detailed physician note* for either cataracts or cataract surgery/surgical procedure for cataract extraction 🡪 case positive.

*Physician notes: Consider notes from ophthalmology and PCP. If no case positive yet, search in discharge summary notes, admission notes, then ED provider notes.

Relevant acronyms:

- OD: oculus dexter (right eye)
- OS: oculus sinister (left eye)
- OU: oculus uterque (both eyes)
- IOL: intraocular lens
- IOLI: intraocular lens implantation
- CE: Cataract extraction
- ECCE: Extracapsular cataract extraction
- ICCE: Intracapsular cataract extraction
- MSICS: Manual small-incision cataract surgery
- SECCE: Small-incision extracapsular cataract extraction surgery
- CEIOL: Cataract extraction with insertion of intraocular lens

*Note: Pseudophakia is most commonly a result of cataract surgery, but in itself (without mention of cataracts or cataract surgery) cannot be used to assume cataracts.*

1. If at end of notes search cannot determine case positive for cataract, look for evidence of cataract surgery/surgical procedure for cataract extraction. Look in surgical department notes as well as procedures for any relevant procedures.

- If there is evidence of a cataract surgical procedure in surgical department notes or procedures, assign case positive. If at this point do not find evidence of cataract surgery, case negative.

**CERVICAL CANCER**

1. Check pathology (including reports specific to the cervix or nearby gynecologic regions). If findings of cervical cancer* 🡪 case positive.

*Terminology/definitions for cervical cancer:

- Cancer terms: cancer/CA, malignancy, malignant neoplasm, carcinoma, metastases of/involving the cervix.
  - Does NOT include terms: benign, dysplasia, metaplasia, carcinoma in situ, or cancer surveillance.
- Cervix terms: cervix/CX, cervix uteri, uterine cervix, endocervix, exocervix, any other parts of cervix.
- Includes both primary cervical cancer or secondary/metastatic (metastases) to cervix.
  - For secondary/metastatic, can be any other cancer with metastasis to/spread to cervix. Or X cancer with cervical involvement (e.g., endometrial carcinoma with cervical stroma invasion/involvement).
  - Does NOT include cancers of other proximal parts of reproductive organs: body of uterus/uterus, endometrium, ovary, vagina unless there is cancer/carcinoma from these organs with cervical involvement/that spreads to cervix (e.g., cervical stroma invasion/involvement).

*Note: Do not consider it cervical cancer if another cancer merely resembles cervical cell histologically. There must be confirmation that cancer in another area of the body originates from or has cancerous cells from the cervix.*

Some histologic types:

- Most common:
  - Squamous cell carcinoma
  - Adenocarcinoma (including adenosquamous)
- Rarer:
  - Adenosquamous and mucoepidermoid carcinoma
  - Adenoid basal carcinoma
  - Carcinoma, unclassifiable
  - Clear cell carcinoma
  - Neuroendocrine or small cell carcinomas
  - Rhabdomyosarcoma of the cervix
  - Primary cervical lymphoma and cervical sarcoma

Staging:

Early-stage cervical cancer refers to FIGO stage IA, IB1, and IB2 disease.

- **Stage IA** – Invasive carcinoma that can be diagnosed only by microscopy, with maximum depth of invasion <5 mm. Stage IA is subdivided into the following categories:
  - **IA1** – Measured stromal invasion ≤3 mm in depth.
  - **IA2** – Measured stromal invasion >3 mm and ≤5 mm in depth.
- **Stage IB1** – Invasive carcinoma with >5 mm depth of stromal invasion, and ≤2 cm in greatest dimension
- **Stage IB2** – Invasive carcinoma >2 cm and ≤4 cm in greatest dimension

1. If no pathology reports or reports normal, check physician notes: 1 detailed physician* note 🡪 case positive.

**See notes above for terminology for cervical cancer.**

*Physician notes: Consider notes from hematology-oncology, radiation oncology, OBGYN (including gynecology-oncology, urogynecology), and PCP. If no case positive yet, search in discharge summary notes, admission notes, then ED provider notes. If at end of notes search do not find evidence for case positive, assign case negative.

**COLON POLYP**

1. Check pathology (reports from tissue sample from colon or rectum generated from colonoscopy procedure) 🡪 search for a report of polyps detected:
   1. Adenomatous (tubular adenoma) (most common)
   2. Hyperplastic polyp
   3. Sessile serrated polyp
   4. Inflammatory polyp
   5. Villous Adenoma
   6. Tubulovillous Adenoma

- Presence of any 1 or more of these polyps is case positive

1. If there are no pathology reports or no mention of above polyps, check physician notes*.
   1. 1 detailed physician note* 🡪 case positive

*Physician notes: Consider notes from gastroenterology and PCP. If no case positive yet, search in discharge summary notes, admission notes, then ED provider notes.

1. If finish notes search without sufficient information to assign case positive, check for any relevant scanned-in outside records that were faxed in. If polyp reported through colonoscopy and/or pathology then case positive. If not, assign case negative.

**DISC DISORDERS**

1. Check imaging. Look for X-ray (XR), CT, MRI/MR, myelogram of the cervical, thoracic, or lumbar spine. Look for evidence of any disc disorders* 🡪 case positive

*Scope of disc disorders include: disc extrusion, protrusion, bulging, desiccation, degeneration/degenerative disc, displacement, loss of disc space, disc height loss, degenerative disc disorder/disease, degenerative discogenic disease, Schmorl’s nodes, disc disorder, intervertebral disc disorder, cervical/thoracic/lumbar disc disorder, intervertebral annulus fibrosus defect (e.g., tear). Look for these terms in cervical, thoracic, or lumbar disc regions (C1-C7, T1-T12, L1-L5, may also see results for L5-S1). Imaging findings/impression sections will often state results by disc level/region, e.g., “disc protrusion at L4-L5” or “degeneration in lower cervical spine.”

1. If no imaging reports or if imaging results are normal, check medication list for presence of relevant medications (see meds list below).
2. If relevant mediations are found, meds plus 1 mention in physician note* 🡪 case positive
3. If no evidence of relevant medication exists, 1 detailed physician note* 🡪 case positive.

**Terminology to look for:** disc extrusion, protrusion, bulging, desiccation, degeneration, displacement, degenerative disc disorder/disease, degenerative discogenic disease, Schmorl’s nodes, disc disorder, intervertebral disc disorder, cervical/thoracic/lumbar disc disorder, intervertebral annulus fibrosus defect. Look for these terms in cervical, thoracic, or lumbar disc regions.

***Physician notes**: Consider notes from PCP, pain management, physical medicine and rehab (PM&R). If no case positive yet, search in discharge summary notes, admission notes, then ED provider notes. If complete notes search and no evidence of case positive, move to step #3.

1. If at end of notes search do not find evidence for case positive, search procedures. Look for diagnostic testing “nerve conduction study” and “EMG” (electromyogram). These tests can pinpoint the location of nerve damage. Evidence of disc disorders 🡪 case positive. If at this point do not find evidence for case positive, assign case negative.

**Medications: muscle relaxers**

- cyclobenzaprine/Flexeril, methocarbamol/Robaxin, carisoprodol/Soma, metaxalone/Metaxall

**DYSMENORRHEA**

1. Search for evidence of treatment in medication list (medication below).
   1. If any treatment exists (oral contraceptive, IUD or NSAID, only one is needed), then treatment plus 1 mention in physician note* 🡪 case positive
   2. If no evidence of treatment/medication exists, 1 detailed physician note* 🡪 case positive.

Other terminology for how condition might be called in diagnosis, notes. These all count:

- Primary dysmenorrhea
- Secondary dysmenorrhea
- Menstrual cramps
- Painful periods/period pain/pain during menses/menstruation (any pain noted as cyclical, that aligns with menses/menstruation)

*Note: In addition to the terminology above, also consider whether symptoms or details are described as occurring during menses/menstruation.*

*Physician notes: Consider notes from OB/GYN and PCP. If no case positive yet, search in discharge summary notes, admission notes, then ED provider notes. If at end of notes search do not find evidence for case positive, assign case negative.

**Medications: oral contraceptive pills (OCPs), NSAIDs (non-steroidal anti-inflammatory drugs), IUDs (intrauterine device)**

Oral contraceptives (OCPs): combined pills (estrogen + progesterone), progesterone only

- Yaz, Yasmin, Ortho-Tri-Cylen Lo, Ortho-Tri-Cylen
- Drospirenone-ethinyl estradiol (Ocella, Yasmin, Zarah, Yaz, [Angeliq](https://www.drugs.com/angeliq.html))
- Norethindrone-ethinyl estradiol (Brevicon, Modicon, Wera, Balziva, Briellyn, Gildagia, Philith, Zenchent)
- Norgestimate-ethinyl estradiol (Estarylla, Previfem, Sprintec, [Femynor](https://www.drugs.com/mtm/femynor.html), [Mili](https://www.drugs.com/mtm/mili.html))
- [Desogestrel](https://www.drugs.com/mtm/ethinyl-estradiol-and-desogestrel.html)-ethinyl estradiol ([Apri](https://www.drugs.com/mtm/apri.html), [Azurette](https://www.drugs.com/mtm/azurette.html), [Caziant](https://www.drugs.com/mtm/caziant.html))
- [Ethynodiol diacetate](https://www.drugs.com/mtm/ethinyl-estradiol-and-ethynodiol-diacetate.html)-ethinyl estradiol ([Kelnor 1/50](https://www.drugs.com/mtm/kelnor-1-50.html), [Zovia 1/35](https://www.drugs.com/mtm/zovia-1-35.html))
- [Levonorgestrel](https://www.drugs.com/mtm/ethinyl-estradiol-and-levonorgestrel.html)-ethinyl estradiol ([Amethyst](https://www.drugs.com/mtm/amethyst.html), [Falmina](https://www.drugs.com/mtm/falmina.html), [Levlen](https://www.drugs.com/mtm/levlen.html))
- [Norgestrel](https://www.drugs.com/mtm/ethinyl-estradiol-and-norgestrel.html)-ethinyl estradiol ([Elinest](https://www.drugs.com/mtm/elinest.html))
- Norelgestromin-ethinyl estradiol
- Etonogestrel-ethinyl estradiol
- [Ethinyl estradiol + drospirenone + levomefolate](https://www.drugs.com/mtm/drospirenone-ethinyl-estradiol-and-levomefolate.html) ([Beyaz](https://www.drugs.com/beyaz.html), [Safyral](https://www.drugs.com/safyral.html))
- [Estradiol + dienogest](https://www.drugs.com/mtm/dienogest-and-estradiol.html) ([Natazia](https://www.drugs.com/natazia.html))
- [norethindrone](https://www.drugs.com/mtm/norethindrone.html) ([Camila](https://www.drugs.com/mtm/camila.html), [Errin](https://www.drugs.com/mtm/errin.html), [Nor-QD](https://www.drugs.com/cdi/nor-qd.html))
- Other ethinyl estradiol combinations

NSAIDs (non-steroidal anti-inflammatory drugs):

- Ibuprofen (Advil, Motrin)
- Naproxen (Aleve, Naprosyn)
- Midol
- Meloxicam (Mobic)
- Diclofenac (Voltaren, Cambia)
- Ketorolac (Toradol)
- Indomethacin (Indocin)
- Celecoxib (Celebrex)

Hormonal IUD (Intrauterine device):

- Levonorgestrel (Mirena, Kyleena, Liletta, Skyla) IUD

**ERECTILE DYSFUNCTION**

1. Check medication list (erectile dysfunction-related medications listed below).
   1. If find medication for erectile dysfunction:
      1. If medication does not belong to PDE5 inhibitor class, assign case positive automatically.
      2. If medication belongs to PDE5 inhibitor class, must also confirm absence of pulmonary hypertension*. Check problem list for pulmonary hypertension/pulmonary arterial hypertension.
         1. If do not find mention of pulmonary hypertension in problem list, assign case positive.
         2. If find mention of pulmonary hypertension in problem list, look for mention of erectile dysfunction in problem list.
            1. If find mention of erectile dysfunction in problem list, assign case positive.
            2. If do not find mention of erectile dysfunction in problem list, cannot assign case positive yet. Need to continue with step #2.
   2. If do not find medication for erectile dysfunction, continue to step #2.

**PDE5 inhibitors can also be used to treat pulmonary hypertension. Therefore, if these medications are being used for case positive, need to confirm that patient does not have pulmonary hypertension, or if they do, that they also have erectile dysfunction.*

1. If no erectile dysfunction medication found or criteria not met for case positive, check for physician note* that discusses condition. 1 detailed physician note* 🡪 case positive

*Physician notes: Consider notes in urology, psychiatry, and PCP. If no case positive yet, search in discharge summary notes, admission notes, then ED provider notes. If complete notes search and do not find evidence for case positive, continue to step #3.

1. If no physician notes or no notes of sufficient detail, search procedure notes and surgical reports for evidence of procedure done for erectile dysfunction (list of procedures below). If evidence of procedure 🡪 case positive. If not, assign case negative.

**Medication:**

- Phosphodiesterase-5 (PDE5) inhibitors: Sildenafil (Viagra, Revatio, Liqrev), vardenafil (Levitra, Staxyn), tadalafil (Cialis, Tadliq), avanafil (Stendra)
- Penile self-injectable drugs (alprostadil or papaverine)
  - Alprostadil brand names: Caverject; Caverject Impulse; Edex; Muse; Prostin VR
- Intraurethral alprostadil

**Procedures:**

- Vacuum devices
- Surgical implementation of penile prosthesis
- Penile revascularization

**HEPATITIS, HEPATOCELLULAR DISEASE**

1. Search imaging. CT, ultrasound (US), MRI/MR, PET scans of the liver, abdomen, or pelvis. If imaging notes hepatitis or hepatocellular disease* 🡪 case positive.

***Qualifying terminology for hepatitis, hepatocellular disease (imaging or notes):**

- (Acute or chronic or unspecified) Hepatitis A (HAV), B (HBV), C (HCV), D (delta), E
- Any other/unspecified acute or chronic hepatitis
- Hepatitis in viral or infectious diseases
- Other hepatitis (alcoholic hepatitis, granulomatous hepatitis, autoimmune hepatitis, toxoplasma hepatitis, secondary syphilitic hepatitis, herpesviral hepatitis, cytomegaloviral hepatitis, mumps hepatitis, nonspecific reactive hepatitis)
- Toxic liver disease with hepatitis
- Sequelae of hepatitis
- Fatty liver (disease)/hepatic steatosis/steatosis of liver
- Steatohepatitis
- Alcoholic fatty liver (disease)
- Alcoholic fibrosis and sclerosis of liver
- Alcoholic liver damage (alcoholic hepatic/liver failure)
- Alcoholic liver disease
- Nonalcoholic steatohepatitis (NASH)
- Nonalcoholic fatty liver (disease) (NAFLD)
- Metabolic dysfunction-associated steatotic liver disease (MASLD)
- Chronic liver disease without mention of alcohol
- Other abnormalities of liver: ascites, fatty accumulation, fatty change, hepatomegaly (hepatic enlargement, enlarged liver), hepatosplenomegaly, dilation of intrahepatic bile ducts.

*Note: Does not include cyst, focus/foci, lesions (unless explicitly labeled as hepatitis/hepatocellular).*

- Abscess of liver
- Portal pyemia
- Hepatic encephalopathy
- Portal hypertension
- Primary sclerosing cholangitis
- Chronic passive congestion of liver
- Other and unspecified disorders/disease of liver (any disease or dysfunction of the liver and intrahepatic bile ducts)
- Sequelae of chronic liver disease
- Wilson’s disease
- Hemochromatosis
- Hepatic infarction/infarction of liver
- Hepatic/liver failure: acute and subacute hepatic failure, alcoholic hepatic failure
- Other and unspecified inflammatory liver diseases
- Central hemorrhagic necrosis of liver
- Acute and subacute necrosis of liver
- Peliosis hepatis
- Hepatic veno-occlusive disease

1. If imaging all normal/no reports available, check physician notes: 1 detailed physician* note 🡪 case positive.

**See notes above for terminology for hepatitis, hepatocellular disease terminology.**

*Note: Do not count mentions of hepatitis solely in context of immunizations.*

*Physician notes: Consider notes from gastroenterology, hepatology, and PCP. If no case positive yet, search in discharge summary notes, admission notes, then ED provider notes. If at end of notes search do not find evidence for case positive, move on to step #3.

1. Check lab results (specific tests below). If any of the following lab results are positive/abnormal/flagged (or if the genotype test provides a genotyping result) 🡪 case positive.
   1. Hepatitis C (HCV):
      1. HCV Quantitative PCR/HCV RNA PCR
      2. HCV genotyping test
   2. Hepatitis B (HBV):
      1. HB surface antigen/HBs Ag (can be own test, or also part of “acute hepatitis panel” test)
      2. HB core antibody/HBc Ab (can be own test, or also part of “acute hepatitis panel” test)
      3. HBV DNA quant/HBV Quant PCR
      4. Hep B E antigen or Hep B E antibody tests
   3. Hepatocellular disease: For these tests, must have 2 abnormal results at least 6 months apart for case positive.
      1. ALT (alanine transaminase) (SGPT): can be a separate test, or part of “hepatic function panel.”
      2. AST (aspartate aminotransferase) (SGOT): can be a separate test, or part of “hepatic function panel.”
   4. Autoimmune hepatitis: antinuclear antibodies (ANA), anti-smooth muscle antibodies, anti-liver/kidney microsomal antibodies, or elevated IgG
   5. Wilson disease: abnormal serum ceruloplasmin
   6. Alpha-1 antitrypsin deficiency: serum alpha-1 antitrypsin level

If, at end of the lab search, there is no evidence for case positive, assign case negative.

**HYPERTHYROIDISM**

1. Case positive can come from:
   1. Medication list alone (list below) **OR**
   2. Surgeries or procedures (list below): Surgery must indicate hyperthyroidism as the underlying condition.
2. If no medications or surgeries/procedures with indication of hyperthyroidism: use physician notes*.
   1. 1 detailed note discussing confirmation of diagnosis or specific diagnosis (see below) plus more detail. Some examples of details include mention of lab monitoring, abnormal lab results (combinations below), or past surgeries/procedures described, etc 🡪 case positive.
      - Acceptable hyperthyroidism diagnoses: hyperthyroidism due to Graves, toxic multinodular goiter, or solitary adenoma
      - Unacceptable diagnoses: hypothyroidism; **acute/transient** or **subclinical** hyperthyroidism e.g., thyroiditis (silent, subacute, postpartum, amiodarone induced), Hashitoxicosis, ingestion of excessive thyroid hormone (e.g., hypothyroidism cases where adjusting medication dose or surreptitious use)

*Physician notes: Consider notes from endocrinology and PCP. If no case positive yet, search in discharge summary notes, admission notes, then ED provider notes. If complete notes search and do not find evidence for case positive, assign case negative.

*Note: Labs alone cannot confirm positive diagnosis. For example, adjusting medication dosage (e.g. levothyroxine) can cause results that appear like hyperthyroidism.*

Labs: combinations of TSH and T4 and/or T3 must occur at the same time point.

- Low TSH and High Free T4 primary hyperthyroidism
- Low TSH and High Free T4 and T3 primary hyperthyroidism
- Low TSH, High T3, normal Free T4 primary hyperthyroidism with T3 toxicosis
- High TSH, High T3, High Free T4 secondary hyperthyroidism from pituitary tumor (central hyperthyroidism, very rare)

Meds: methimazole, propylthiouracil (PTU)

Surgeries, procedures:

- Radioactive iodine (RAI): check nuclear medicine imaging, procedures
- Radioactive iodine uptake test (RAIU): check nuclear medicine imaging, procedures
- Surgical removal of thyroid (thyroidectomy): check surgical procedure notes, procedures. Also, check the pathology report of thyroid tissue.
- Surgical complications related to hyperthyroidism: check surgical procedure notes, procedures.

**INTERSTITIAL CYSTITIS**

1. Search for evidence of treatment in medication list (medications below).
2. If treatment exists, then treatment plus 1 mention in physician note* 🡪 case positive
3. If no evidence of treatment/medication exists, 1 detailed physician note* 🡪 case positive.

Other terminology for how condition might be called in diagnosis, notes:

- interstitial cystitis (IC), painful bladder syndrome (PBS), bladder pain syndrome (BPS)

*Note: Does NOT include acute, chronic, or unspecified cystitis without explicit mention of* interstitial *cystitis or its synonyms*

*Does NOT include unspecified bladder symptoms, unspecified pelvic pain, pelvic floor dysfunction, urinary tract infection (UTI).*

*Symptoms of interstitial cystitis (e.g., urinary urgency and/or frequency, pain, pressure, or tenderness in the pelvic or bladder area, pain during sex) can be related to many conditions, so unless note explicitly mentions interstitial cystitis or its synonyms, do not consider.*

*Physician notes: Consider notes from urology, female pelvic medicine and pelvic reconstructive surgery, OB/GYN (including urogynecology), and PCP. If no case positive yet, search in discharge summary notes, admission notes, then ED provider notes. If at end of notes search do not find evidence for case positive, assign case negative.

**Medications:**

- Pentosan polysulfate sodium (Elmiron), pentosan polysulfate (Elmiron)

**LIVER CANCER**

1. Check pathology (relevant reports can be specific to liver or include other regions as well). If findings of liver cancer* 🡪 case positive.
2. If no pathology reports or pathology normal, check imaging (CT, ultrasound (US), MRI/MR, PET of liver, abdomen, abdomen). If imaging reports abnormalities in the liver (e.g., foci, lesions) and includes concern, suspicion, or confirmation of cancer or metastatic disease 🡪 case positive. Or if imaging confirms cancer/metastatic disease 🡪 case positive.

Terminology/definitions for liver cancer:

- Cancer terminology: cancer/CA, malignancy, malignant neoplasm, carcinoma, metastases of/involving the liver.
  - Does NOT include terms: benign, dysplasia, metaplasia, carcinoma in situ, or cancer surveillance. Does not include liver hemangioma.
- Liver terminology: liver, intrahepatic bile ducts, hepatic, hepatocellular.
  - Does NOT include bile duct cancer/cancer of extrahepatic bile ducts (only include if specified as intrahepatic bile duct)
- Other terminology: hepatocellular carcinoma (HCC), hepatoblastoma, angiosarcoma of liver, other sarcomas of liver.
- Cancer in liver includes primary or secondary/metastatic (metastases).
  - For secondary/metastatic, can be any other cancer with metastasis to/spread to liver. Or another primary cancer with documented liver involvement or metastases to the liver.

1. If imaging and pathology all normal/no reports, check physician notes: 1 detailed physician* note 🡪 case positive.

**See notes above for terminology for liver cancer.**

*Physician notes: Consider notes from hematology-oncology, radiation oncology, and PCP. If no case positive yet, search in discharge summary notes, admission notes, then ED provider notes. If at end of notes search do not find evidence for case positive, assign case negative.

**OSTEOPOROSIS**

1. Check imaging (DEXA or nuclear medicine (NM) Bone Mineral Density Report) 🡪 if report of osteoporosis (T-score $\leq$ -2.5 usually at hip or lumbar spine), then case positive. *Note: for lumbar spine, L1-L4 average T-score on DEXA must be* $\leq$ *-2.5 for a case positive. A T-score* $\leq$ *-2.5 at any single lumbar location is not sufficient alone for a case positive.*
2. If DEXAs are normal or not present *(or if T-score indicates normal/osteopenia, which has T-score -1.0 to -2.4)*, search for evidence of osteoporosis treatment in the medication list (medications below).
   1. If treatment is documented, treatment plus 1 mention in physician note* discussing osteoporosis 🡪 case positive.
   2. If no treatment is documented, but there is 1 detailed physician note* discussing osteoporosis 🡪 case positive.

*Note: mention of insufficiency fracture is not sufficient to assume osteoporosis without specific mention/discussion of osteoporosis as the risk factor.*

*Physician notes: Consider notes from PCP and endocrinology. If no case positive yet, search in discharge summary notes, admission history and physical notes, then ED provider notes.

1. If neither 2a nor 2b is met after full note review, check for any relevant outside scanned records (e.g., faxed DEXA reports). If osteoporosis reported through DEXA (T-score $\leq$ -2.5 usually at hip or lumbar spine) then case positive. If not, assign case negative.

**Medications**:

- oral bisphosphonates (alendronate, risedronate) first-line therapy
- denosumab in people intolerant or incompletely responsive to bisphosphonate, or CKD stage IV
- zoledronate IV in people where oral bisphosphonates contraindicated (CKD stage IV or esophageal disease)

**OTHER CANCERS, NEOPLASMS OF UNSPECIFIED NATURE**

1. Start with physician notes: 1 detailed physician* note 🡪 case positive.

**Terminology/scope of other cancers, neoplasms of unspecified nature:**

- This condition category includes:
  - Primary or secondary (metastatic) cancer of any part of body (that are not otherwise specified as MWI oncologic conditions), including head/face/neck, respiratory organs, GI organs, reproductive and urinary organs, bones, glands, bone marrow, connective and soft tissue, lymphoid and hematopoietic tissue, lymph nodes, skin.
  - Neoplasms of unspecified nature or of uncertain/unspecified behavior of any part of body (NUB) from above (besides skin).
  - Other relevant terms for cancers and neoplasms include cancer, CA, malignancy, malignant neoplasm, carcinoma, sarcoma, neoplasm, tumor, metastases (mets) to/involving (e.g., with lung or other organ involvement).
  - Does not include neoplasms explicitly noted as benign.

*Physician notes: Consider notes from hematology-oncology, surgical oncology, and PCP. If no case positive yet, search in discharge summary notes, admission notes, then ED provider notes. If at end of notes search do not find evidence for case positive, move on to step #2.

1. If nothing in notes, search all pathology reports. If reports note applicable terminology (see above), case positive.
2. If no pathology reports/reports normal, search imaging. If reports note applicable terminology (see above), case positive. If, after searching through imaging reports, there is no evidence of case positive, assign case negative.

**OTHER NEUROLOGIC DISORDERS**

1. Start with physician notes: 1 detailed physician* note 🡪 case positive.

**Terminology for neurologic disorders:**

- Neuropathy, polyneuropathy
- Aphasia
- Dysphasia
- Anoxic brain damage, hypoxia of brain
- Mild cognitive impairment, mild neurocognitive disease/mild cognitive disorder
- Cerebral degeneration
- Huntington’s chorea
- Other choreas (including drug-induced)
- Neuroleptic malignant syndrome
- Congenital nonprogressive ataxia
- Friedreich’s ataxia
- Hypomyelination-hypogonadotropic hypogonadism-hypodontia
- Leukodystrophy with vanishing white-matter disease
- Other and unspecified hereditary ataxias
- Primary cerebellar degeneration
- Other cerebellar ataxia
- Other and unspecified spinocerebellar diseases
- Werdnig-Hoffmann disease (infantile spinal muscular atrophy type I)
- Spinal muscular atrophy, including Kugelberg-Welander disease, inherited, unspecified
- Subacute combined degeneration of spinal cord in diseases elsewhere
- Neuromyelitis optica
- Acute and subacute hemorrhagic leukoencephalitis
- Other and unspecified acute disseminated demyelination
- Other demyelinating diseases of CNS
- Schilder’s disease
- Acute (transverse) myelitis, idiopathic transverse myelitis
- Systemic atrophies primarily affecting the central nervous system in diseases classified elsewhere, including paraneoplastic neuromyopathy and neuropathy.
- Secondary parkinsonism (malignant neuroleptic syndrome, drug-induced, neuroleptic induced, other agents, postencephalitic, vascular, other and unspecified secondary parkinsonism)
- Degeneration of nervous system due to alcohol
- Alpers disease
- Leigh disease
- Corticobasal degeneration
- Alexander disease
- Other and unspecified degenerative diseases of nervous system (including degeneration of spinal cord)
- Other and unspecified demyelinating diseases of central nervous system
- Unspecified disorders of nervous system
- Huntington’s disease
- Does not include amyotrophic lateral sclerosis (ALS) or motor neuron diseases (MND), stroke/CVA/cerebrovascular accident/cerebrovascular disease including transient ischemic attack (TIA), dementia including Alzheimer’s, migraine headache, multiple sclerosis (MS), paralytic syndrome, Parkinson disease (except YES include secondary parkinsonism), restless legs syndrome (RLS), and seizure disorder/epilepsy. Does not include isolated encephalopathy.

*Physician notes: Consider notes from neurology, neurological surgery, and PCP. If no case positive yet, search in discharge summary notes, admission notes, then ED provider notes. If at end of notes search do not find evidence for case positive, assign case negative.

**PANCREATITIS**

1. Check imaging (CT, ultrasound/US, MRI/MR, MRCP of: abd/abdomen, pelvis, pancreas). Look for evidence of pancreatitis* 🡪 case positive

*Look for any of the following descriptions of the pancreas: presence of calcifications, dilated pancreatic duct/ductal dilation, atrophy/atrophic pancreas. These can be with or without explicit mention of pancreatitis or chronic pancreatitis (imaging reports are often not accompanied by explicit mention of pancreatitis or chronic pancreatitis—that is OK for case positive). Confirm no explicit diagnosis of acute pancreatitis in the report (in which case do not consider as case positive and continue the chart review).

1. If no imaging/imaging results normal, check procedures: ERCP (Endoscopic retrograde cholangiopancreatography), upper GI endoscopy, endoscopy reports. Look for pancreatitis using same criteria as Step 1* 🡪 case positive
2. If no procedure reports/procedures are normal, check notes. 1 detailed physician note* for pancreatitis**🡪 case positive

*Physician notes: Consider notes from gastroenterology, pain management, and PCP. If no case positive yet, search in discharge summary notes, admission notes, then ED provider notes. If at end of notes search do not find evidence for case positive, assign case negative.

**For our purposes, scope of pancreatitis includes pancreatitis unspecified as to whether acute or chronic, chronic pancreatitis (including acute on chronic), cytomegaloviral pancreatitis, alcohol induced chronic pancreatitis. However, does NOT include acute pancreatitis. Therefore, for notes, need mention of pancreatitis/chronic pancreatitis and details (e.g., etiology, prior episodes, imaging/lab findings, management).

- If we see diagnosis of acute pancreatitis plus details 🡪 do not consider, continue notes search.
- If see diagnosis of any chronic pancreatitis plus details 🡪 case positive.
- If we see diagnosis of “pancreatitis” (aka unspecified if acute/chronic) and details 🡪 check rest of note for additional mention elsewhere of diagnosis of acute pancreatitis. If there is no mention elsewhere of acute pancreatitis 🡪 case positive. If acute pancreatitis is diagnosed elsewhere in same note 🡪 do not consider, continue notes search.

**PEPTIC ULCER**

1. Check procedures (often labelled upper gastrointestinal endoscopy/endoscopy). Clinical impression of peptic ulcer* on procedure report 🡪 case positive.

*For our purposes, scope of peptic ulcer includes esophagus, gastric (stomach), duodenal, peptic, gastrojejunal ulcers. Reports may also mention erosions (e.g. gastric erosion). Both ulcers and erosions in any of these areas count for case positive. *Other location terms may also be used, especially in case of stomach e.g. antrum, proximal body, pre-pyloric or pyloric region. These all count as case positive.*

*Note: Condition scope does not include varices such as esophageal varices.*

1. If procedure reports are absent or do not mention peptic ulcer or equivalent terms in these reports, check for physician notes* that discuss condition.
   1. 1 detailed physician note* 🡪 case positive

*Physician notes: Consider notes from gastroenterology and PCP. If no case positive yet, search in discharge summary notes, admission notes, then ED provider notes. If at end of notes search do not find evidence for case positive, assign case negative.

**PREMENSTRUAL SYNDROME (PMS), PREMENSTRUAL DYSPHORIC DISORDER (PMDD)**

1. Search for evidence of treatment in medication list (medication below).
   1. If any treatment exists. Only one (oral contraceptive or antidepressant) is sufficient, then treatment plus 1 mention in physician note* 🡪 case positive
   2. If no evidence of treatment/medication exists, 1 detailed physician note* 🡪 case positive.

Other terminology for how condition might be called in diagnosis, notes. These all count:

- Premenstrual tension syndrome (PMT)
- Mittelschmerz/ovulation pain/ovulatory pain

*Note: In addition to terminology above, can also consider if there is discussion of symptoms/details in correct timing (pre) in relation to menses/menstruation.*

*Physician notes: Consider notes from OB/GYN and PCP. If no case positive yet, search in discharge summary notes, admission notes, then ED provider notes. If complete notes search and do not find evidence for case positive, assign case negative.

**Medications: oral contraceptives, antidepressants**

Oral contraceptives: combined pills (estrogen + progesterone), progesterone only

- Yaz, Yasmin, Ortho-Tri-Cylen Lo, Ortho-Tri-Cylen
- Drospirenone-ethinyl estradiol (Ocella, Yasmin, Zarah, Yaz, [Angeliq](https://www.drugs.com/angeliq.html))
- Norethindrone-ethinyl estradiol (Brevicon, Modicon, Wera, Balziva, Briellyn, Gildagia, Philith, Zenchent)
- Norgestimate-ethinyl estradiol (Estarylla, Previfem, Sprintec)
- [Desogestrel](https://www.drugs.com/mtm/ethinyl-estradiol-and-desogestrel.html)-ethinyl estradiol ([Apri](https://www.drugs.com/mtm/apri.html), [Azurette](https://www.drugs.com/mtm/azurette.html), [Caziant](https://www.drugs.com/mtm/caziant.html))
- [Ethynodiol diacetate](https://www.drugs.com/mtm/ethinyl-estradiol-and-ethynodiol-diacetate.html)-ethinyl estradiol ([Kelnor 1/50](https://www.drugs.com/mtm/kelnor-1-50.html), [Zovia 1/35](https://www.drugs.com/mtm/zovia-1-35.html))
- [Levonorgestrel](https://www.drugs.com/mtm/ethinyl-estradiol-and-levonorgestrel.html)-ethinyl estradiol ([Amethyst](https://www.drugs.com/mtm/amethyst.html), [Falmina](https://www.drugs.com/mtm/falmina.html), [Levlen](https://www.drugs.com/mtm/levlen.html))
- [Norgestimate](https://www.drugs.com/mtm/ethinyl-estradiol-and-norgestimate.html)-ethinyl estradiol ([Estarylla](https://www.drugs.com/mtm/estarylla.html), [Femynor](https://www.drugs.com/mtm/femynor.html), [Mili](https://www.drugs.com/mtm/mili.html))
- [Norgestrel](https://www.drugs.com/mtm/ethinyl-estradiol-and-norgestrel.html)-ethinyl estradiol ([Elinest](https://www.drugs.com/mtm/elinest.html))
- Norelgestromin-ethinyl estradiol
- [Ethinyl estradiol + drospirenone + levomefolate](https://www.drugs.com/mtm/drospirenone-ethinyl-estradiol-and-levomefolate.html) ([Beyaz](https://www.drugs.com/beyaz.html), [Safyral](https://www.drugs.com/safyral.html))
- [Estradiol + dienogest](https://www.drugs.com/mtm/dienogest-and-estradiol.html) ([Natazia](https://www.drugs.com/natazia.html))
- [norethindrone](https://www.drugs.com/mtm/norethindrone.html) ([Camila](https://www.drugs.com/mtm/camila.html), [Errin](https://www.drugs.com/mtm/errin.html), [Nor-QD](https://www.drugs.com/cdi/nor-qd.html)).
- Any other ethynyl estradiol combinations.

Antidepressants:

- SSRIs: Fluoxetine (Prozac), sertraline (Zoloft), citalopram (Celexa), escitalopram (Lexapro), paroxetine (Paxil), fluvoxamine
- SNRIs: venlafaxine (Effexor), duloxetine (Cymbalta)
- Atypicals: Bupropion/Wellbutrin, mirtazapine/Remeron
- Ketamine (newer, less common)
- Less common: serotonin modulators (trazodone), TCAs (amitriptyline, imipramine, nortriptyline), MAO inhibitors (phenelzine)

**RESTLESS LEGS SYNDROME (RLS)**

1. Search for evidence of treatment in medication list (medication below).
2. If treatment exists, then treatment plus 1 mention in physician note* 🡪 case positive
3. If no evidence of treatment/medication exists, 1 detailed physician note* 🡪 case positive.

*Note:* *Do not count periodic limb movement disorder (PLMD) as RLS. Although related, it is outside the scope of RLS.*

*Physician notes: Consider notes from neurology, sleep medicine, neurology-sleep program, pulmonary, and PCP. If no case positive yet, search in discharge summary notes, admission notes, then ED provider notes. If at end of notes search do not find evidence for case positive, assign case negative.

**Medications:**

- Gabapentin, gabapentin enacarbil, levodopa, methadone, oxycodone, oxycodone-naloxone (e.g., Targiniq, Troxyca), pramipexole, pergolide, pregabalin, ropinirole, rotigotine

**SUBSTANCE USE DISORDERS (SUDs), INCLUDING ALCOHOL**

1. Check laboratory results (urine drug screen, blood alcohol level (“alcohol, ethyl”), and GGT (gamma-glutamyl transferase) tests). Check medication list (see list of medications below).
   1. If results of lab abnormal or presence of medication for SUDs exists, then that plus 1 mention in physician note* 🡪 case positive
   2. If no evidence of medication/abnormal lab, 1 detailed physician note* 🡪 case positive.

**Terminology for substance use disorders:**

- Alcohol (EtOH) intoxication, poisoning, use (use/consumption), abuse, dependence, withdrawal, alcoholism/history of alcoholism
- Drug use (use/consumption), abuse, dependence, withdrawal
  - Types of drugs: opioids, sedatives, hypnotics, anxiolytics, cocaine, cannabis (marijuana), amphetamine and other psychostimulants, hallucinogen, inhalant, antidepressant, other or unspecified drugs/substances/narcotics, combinations of drugs
    - E.g., benzodiazepines
  - *Note: Nicotine/tobacco use is not included unless in combination with another substance listed above*
- Alcohol or drug induced mental disorders: delirium, amnestic disorder, dementia, psychotic disorder, intoxication, withdrawal, anxiety disorder, sexual dysfunction, mood or sleep disorder, among others.
- Alcoholic polyneuropathy
- Alcoholic gastritis
- Toxic effects of alcohol
- Pellagra/niacin deficiency
- Counseling or surveillance for alcohol or drug/substance use and abuse.

*Note: Given the high prevalence of alcohol consumption, do not consider documentation such as ‘”X number of drinks/week” or “patient consumes alcohol” in the social history section. Goal to determine if substance use/consumption is “notable”. Terms “significant” or “notable” or “chronic” or “heavy user” or “daily,” history/details on past/present use and quantity are considered details and can also be indicative of use disorder. Additionally, terms such as “now sober/has quit,” “trying to quit/cut back” also stronger indicators of SUDs.*

*Physician notes: Consider notes from PCP, psychiatry, and addiction medicine. If no case positive yet, search in discharge summary notes, admission notes, then ED provider notes. If at end of notes search do not find evidence for case positive, assign case negative.

**Medications:**

- Naloxone, methadone, buprenorphine
- For alcohol: naltrexone, acamprosate, gabapentin
- For cannabis: N-acetylcysteine (NAC), topiramate, gabapentin, nabiximols, cannabidiol, varenicline (Chantix)
